# Effectiveness of direct oral anticoagulants for all‐cause mortality and cardiovascular events in overweight and obese patients with atrial fibrillation: Insight from the nationwide START registry

**DOI:** 10.1111/eci.70148

**Published:** 2025-11-13

**Authors:** Danilo Menichelli, Gianluca Gazzaniga, Emilia Antonucci, Gualtiero Palareti, Daniela Poli, Pasquale Pignatelli, Daniele Pastori, the START2 Register Investigators, Benilde Cosmi, Daniela Poli, Elena Lotti, Martina Berteotti, Walter Ageno, Giovanna Colombo, Salvatore Bradamante, Eugenio Bucherini, Monica Vastola, Paola Casasco, Antonio Chistolini, Alessandra Serrao, Luciano Crippa Ambulatorio Emostasi, Raimondo De Cristofaro, Erica De Candia, Valeria De Micheli, Marcello Di Nisio, Anna Falanga, Teresa Lerede, Elvira Grandone, Donatella Colaizzo, Antonio Insana, Nicola Lucio Liberato, Domenico Lione, Rosa Maria Lombardi, Giacomo Lucarelli, Giuseppe Malcangi, Catello Mangione, Giuliana Martini Centro Emostasi, Marco Marzolo, Giovanni Nante, Vincenzo Oriana, Paolo Pedico, Simona Pedrini, Vittorio Pengo, Antonietta Piana, Francesco Cibecchini, Simona Pezzella, Pasquale Pignatelli, Daniele Pastori, Vincenza Rossi, Lucia Ruocco, Paolo Chiarugi, Serena Rupoli, Domizio Serra, Carmine Spataro, Margherita Reduzzi, Sophie Testa, Oriana Paoletti, Vincenzo Toschi, Claudio Vasselli

**Affiliations:** ^1^ Department of General Surgery, Surgical Specialty and Anesthesiology Paride Stefanini Sapienza University of Rome Rome Italy; ^2^ Arianna Anticoagulazione Foundation Bologna Italy; ^3^ Department of Critical Care Medicine, Thrombosis Centre Azienda Ospedaliera Universitaria Careggi Firenze Italy; ^4^ Department of Medical and Cardiovascular Sciences Sapienza University of Rome Rome Italy; ^5^ IRCCS Neuromed, Località Camerelle Pozzilli Isernia Italy

**Keywords:** atrial fibrillation, BMI, DOAC, obesity, overweight, VKA

## Abstract

**Background:**

Overweight and obesity are highly prevalent in atrial fibrillation (AF) patients, yet the best anticoagulant strategy for this group is still unclear. We evaluated the risk of all‐cause mortality and cardiovascular events (CVEs) in overweight and obese AF patients treated with direct oral anticoagulants (DOACs) or vitamin K antagonists (VKAs).

**Methods:**

We analysed 10,259 AF patients on anticoagulants from the prospective nationwide START registry. Overweight was defined as BMI 25–29.9 kg/m^2^ and obesity as BMI ≥30 kg/m^2^. Mortality risk was assessed with Cox proportional hazards models, and CVEs with Fine–Grey models accounting for competing risks. Additional modelling strategies, subgroup analyses, and propensity score matching were performed to explore data and ensure robustness.

**Results:**

Overall, 6534 (63.7%) patients had BMI >25 kg/m^2^ (65.7% overweight, 34.3% obese). Over a median follow‐up of 17.4 months, 408 deaths and 481 CVEs occurred. DOACs' use was associated with a lower risk of all‐cause mortality (HR 0.57, 95% CI 0.46–0.70, *p* < 0.001) and CVEs (sHR 0.71, 95% CI 0.59–0.86, *p* < 0.001) versus VKAs. The inverse association of DOACs with mortality was evident in both overweight (HR 0.52, 95% CI 0.39–0.68, *p* < 0.001) and obese patients (HR 0.69, 95% CI 0.48–0.98, *p* = 0.040), while the reduction in CVEs reached significance only in the overweight group.

**Conclusions:**

In this nationwide registry, DOACs were associated with lower risks of mortality and CVEs compared with VKAs across BMI groups, though the CVE effect was not evident in obese patients. These findings suggest a potential mortality advantage of DOACs irrespective of BMI, warranting further confirmation.

## Graphical abstract

This study shows that overweight and obese patients with atrial fibrillation experience improved outcomes when treated with DOACs compared with VKAs. Specifically, DOAC use was associated with lower risks of all‐cause mortality and cardiovascular events. The mortality benefit was consistent across both overweight and obese groups, while the reduction in cardiovascular events was more evident in overweight patients. These findings suggest a mortality advantage of DOACs irrespective of BMI and support their preferential use in this population.

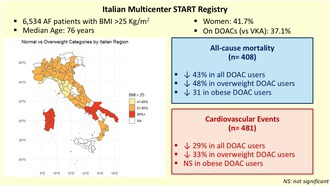

## 1 INTRODUCTION

Obesity affected around 890 million adults worldwide in 2022, corresponding to 16.0% of adults ≥18 years worldwide.^1^ The prevalence of obesity has been increasing worldwide in the last decades and, according to the Global Burden of Disease, it is estimated to affect 2.1 billion adults in 2013.^2^ In particular, the prevalence of obesity raised up to 50% of the adult population in some countries from Oceania, North Africa, and the Middle East,^3^ while it was about 41.8% in North America^1^ and 20% in Western Europe.^3^ In particular, a low prevalence (<20%) of obesity was observed in countries from the Mediterranean area that had an obesity prevalence between 20.0% and 24.9%,^4^ while Northern (i.e. United Kingdom and Ireland), and Eastern (i.e. Bulgaria, Hungary and Lithuania) Europe had a higher prevalence of obesity (25.0%–29.9%).^4^ In the past decades, the prevalence of obesity in Italy was 8%, due to a healthy diet and lifestyle; however, in the last years, the prevalence has increased abruptly by 27.5% due to lifestyle changes such as the consumption of high‐caloric foods and sedentary behaviors^5^ reaching 19.9% in 2019.^4^

It was estimated that the rising of the overweight and obese population provoked 3.4 million deaths and 3.9% of years of life lost worldwide.^2^ In addition, obesity has been associated with several cardiovascular (CV) risk factors such as arterial hypertension, diabetes mellitus and dyslipidemia^3^ and CV disease.^3^ Among CV diseases, atrial fibrillation (AF) is strongly associated with obesity^6^: indeed, epidemiologic studies showed that the two major predictors of new‐onset AF are arterial hypertension and obesity.^6^, ^7^ In particular, data from the Framingham Heart Study, seem to suggest that obesity could increase the risk of AF by 50%.^8^ Several mechanisms such as epicardial adiposity, inflammation, oxidative stress, fibrosis, ion channel alterations, and autonomic dysfunction seem to be involved in the left atrium enlargement and AF pathogenesis in obese patients.^6^

Although obesity is a risk factor for new‐onset AF, its role in the prognosis of AF patients is still contradictory. Indeed, results from a post‐hoc analysis of The Effective Anticoagulation With Factor Xa Next Generation in Atrial Fibrillation–Thrombolysis in Myocardial Infarction 48 (ENGAGE AF‐TIMI 48) trial revealed that overweight and obese patients with AF had a lower risk of stroke/systemic embolism and mortality.^9^ These results were coherent with a post‐hoc analysis of the Atrial Fibrillation Follow‐up Investigation of Rhythm Management (AFFIRM) trial, in which obese and overweight patients had a lower all‐cause mortality risk compared to normal weight.^10^ Conversely, data from the prospective Danish Diet, Cancer and Health study, in which 3135 AF patients were enrolled and followed up to 4.9 years found a direct association between overweight/obesity and ischemic stroke, thromboembolism or death.^11^ Also, a large meta‐analysis including 49,364 AF patients found no difference in all‐cause and CV death risk between obese and normal weight AF patients.^12^

While long‐term anticoagulation treatment with vitamin K antagonists (VKAs) or direct oral anticoagulants (DOACs) is effective in the majority of AF patients to reduce thromboembolism,^13^ less evidence is available in patients with obesity. In previous studies^14^, ^15^, ^16^, ^17^, ^18^ the effectiveness and safety of DOAC in obese patients with AF appear controversial.

In addition, few prospective studies based on real‐world data from observational studies are available. For this reason, the aim of our study was to investigate the effectiveness of DOACs compared to VKAs in a real‐world cohort of overweight and obese patients with AF.

## 2 METHODS

### 2.1 START Registry

The START registry is a prospective, observational, multicenter cohort study conducted in Italy, enrolling patients aged 18 years or older who initiate anticoagulation therapy for AF. Comprehensive details about the registry have been previously published.^19^ Briefly, inclusion criteria comprised adult patients with AF requiring oral anticoagulation; patients receiving low‐molecular‐weight heparin were excluded. Individuals with a life expectancy of less than 6 months, those not residing in the participating region, or planning to relocate within the next 6 months were not eligible for inclusion in the registry. Patients already participating in phase 2 or 3 clinical trials were also excluded, whereas those enrolled in other observational or phase 4 studies were eligible.

The study is registered at ClinicalTrials.gov (NCT02219984) and received approval from the Institutional Review Boards of all participating institutions. Informed consent was obtained from each participant prior to inclusion, and the study protocol complies with the ethical standards set forth in the 1975 Declaration of Helsinki.

This post‐hoc analysis was restricted to only patients with AF and overweight or obesity.

### 2.2 Baseline features

Baseline variables comprised demographic information and clinical data, encompassing cardiovascular risk factors, routinary laboratory parameters, relevant comorbidities, left ventricular ejection fraction, smoking status, indication for anticoagulation therapy, the specific class of oral anticoagulant administered, and concurrent pharmacological treatments. A history of cardiovascular disease was defined as documented coronary artery disease (CAD), including prior myocardial infarction or coronary revascularization (via percutaneous coronary intervention or coronary artery bypass grafting). Cerebrovascular disease was defined by a prior ischemic stroke or transient ischemic attack (TIA).

### 2.3 Overweight and obesity definitions

Obesity and overweight were defined and classified according to The World Health Organization (WHO).^4^, ^20^, ^21^ The WHO,^21^ classifies adult obesity using the body mass index (BMI), which is measured by calculating [(weight in kg)/(height in m)^2^]. Overweight was defined by BMI between 25.0 and 29.9 kg/m^2^ and obesity by a BMI ≥30.0 kg/m^2^. Obese patients were stratified into class I (BMI: 30.0–34.9 kg/m^2^); class II (BMI: 35.0–39.9 kg/m^2^) and class III (BMI ≥40.0 kg/m^2^).

### 2.4 Study Endpoints

The aim of this study was to investigate the association between oral anticoagulant type (DOACs vs. VKAs) and clinical outcomes in patients with a BMI >25 kg/m^2^, with particular attention to differences between overweight and obese subgroups. Specifically, all‐cause mortality and the occurrence of cardiovascular events (CVEs) were evaluated according to anticoagulant type, both in the overall population, and according to overweight and obesity. CVEs were defined as fatal or non‐fatal myocardial infarction, coronary revascularization, fatal or non‐fatal ischemic stroke, and transient ischemic attack. All events were prospectively documented during follow‐up visits and recorded by registry investigators in the electronic case report form.

### 2.5 Statistical analysis

Descriptive statistics were employed to characterize the study population. The distribution of continuous variables was evaluated using the Kolmogorov–Smirnov test. Variables with normal distribution were summarized using means and standard deviations, whereas non‐normally distributed variables were expressed as medians with interquartile ranges (IQR). Categorical variables were reported as absolute frequencies and percentages. Group comparisons for continuous variables were performed using the independent samples *t*‐test or the Wilcoxon rank‐sum test, depending on the distribution. Categorical variables were compared using the chi‐square test or Fisher's exact test, as appropriate. For comparisons involving more than two groups, one‐way analysis of variance (ANOVA) or the non‐parametric Kruskal–Wallis test was applied.

To explore the geographical distribution of patients with BMI >25 kg/m^2^, the population was stratified by Italian region, and a choropleth map was produced to visualize regional differences.

To evaluate differences in all‐cause mortality based on anticoagulant type, we conducted Kaplan–Meier survival analyses in the overall study population. Survival curves were generated using the Kaplan–Meier method and compared using the log‐rank test. Univariable and multivariable Cox proportional hazards regression models were subsequently used to estimate hazard ratios (HRs) and corresponding 95% confidence intervals (CIs) for all‐cause mortality associated with the use of DOACs vs. VKAs and individual clinical variables.

The Fine–Grey subdistribution hazard model was employed to estimate the cumulative incidence of CVEs, accounting for the competing risk of all‐cause mortality. Cumulative Incidence Functions (CIF) were plotted to visualize cumulative incidence.

The following clinical and pharmacological variables were evaluated as candidate predictors in both Cox proportional hazards and Fine–Grey competing risks regression models: use of DOAC, BMI category, age ≥75 years, female sex, history of hypertension, diabetes mellitus, CAD, anaemia, impaired renal function (expressed as estimated glomerular filtration rate – eGFR), heart failure (HF), active smoking, previous stroke or TIA, peripheral artery disease (PAD), chronic obstructive pulmonary disease or obstructive sleep apnoea syndrome (COPD/OSAS), antiplatelet therapy, use of class 1c antiarrhythmic drugs, amiodarone, lipid‐lowering therapy, renin–angiotensin–aldosterone system inhibitors (RAASi), beta blockers, calcium channel blockers, diuretics, digoxin, and proton pump inhibitors (PPI). Variables demonstrating statistical significance in univariable analyses (*p* < 0.05) were subsequently included in the multivariable models.

These analyses were initially conducted in the overall population of patients with a BMI greater than 25 kg/m^2^ and subsequently repeated after stratifying the cohort by overweight and obese categories.

To avoid potential bias from variable selection based only on univariate p‐values filtering, we constructed a second multivariable Cox model and Fine–Grey model including a set of prespecified clinically relevant covariates (i.e. anticoagulant type, BMI class, age, sex, hypertension, diabetes, CAD, anaemia, renal function [eGFR], HF, Paroxysmal Atrial Fibrillation, PAD, and COPD/OSAS). To formally test for effect modification by BMI, the models also included an interaction term between anticoagulant type and BMI class.

The proportional hazards assumption was assessed using Schoenfeld residuals (data shown in Table S1).

To address potential confounding between patients treated with DOACs and those receiving VKAs, we used propensity score matching (PSM) to evaluate the consistency of results across modelling approaches. Propensity scores were estimated via logistic regression, including the following predefined covariates: sex, age, HF, BMI, hypertension, diabetes, CAD, anaemia, eGFR, previous stroke/TIA, COPD/OSAS, smoking, antiplatelet, Class 1c AAD, amiodarone, and beta‐blockers' use. These variables were selected as they represent the most frequent comorbidities and treatments in patients with AF. DOAC users were matched 1:1 to VKA users using nearest‐neighbour matching with a calliper width of 0.1, without replacement. Covariate balance after matching was assessed with standardized mean differences (SMDs), with values <0.1 indicating adequate balance. Patient characteristics before and after matching are summarized in Table S2, and additional PSM balance diagnostics are reported in Table S3. The matched cohorts were subsequently analysed. HR for all‐cause mortality was derived from a univariate Cox proportional hazards model, while the risk of CVEs was evaluated using a univariate Fine–Grey model, accounting for all‐cause death as a competing risk.

Lastly, we performed some exploratory analyses. (1) Restricted Cubic Splines (RCS) with 4 default knots were used to assess potential non‐linear associations between BMI, treated as a continuous variable, and all‐cause mortality in a univariable model. (2) Patients were stratified according to time in therapeutic range (TTR), calculated using the Rosendaal method,^22^ with 60% as the threshold; patients with missing TTR values were excluded from this analysis. (3) An analysis to compare the use of DOAC vs. VKA in each subgroup of patients according to obesity severity (I‐III) with overweight as a reference. (4) A subgroup analysis for all‐cause mortality and CVE risk was conducted in patients weighing ≥100 kg. (5) Univariable Cox and Fine‐Grey models were used to examine the association of each DOAC agent versus VKA with all‐cause mortality and CVEs, respectively.

Statistical analyses were conducted using R (version 4.2.3) and IBM SPSS Statistics (version 25.0), with a two‐sided significance threshold set at *α* = 0.05.

## 3 RESULTS

### 3.1 Geographic distribution

Overall, 10,259 patients were enrolled in the START registry. Of these, 6534 patients (63.7%) had a BMI >25 kg/m^2^. The geographic distribution of patients with BMI >25 kg/m^2^ across Italian regions is shown in Figure S1. The highest prevalence of BMI >25 kg/m^2^ relative to total patients was observed in Puglia (655 patients, 81.1%), followed by Calabria (75%) regions.

### 3.2 Descriptive analysis

Table 1 summarizes the baseline characteristics of the 6534 patients with BMI >25 kg/m^2^ enrolled in the START registry, stratified by overweight (BMI 25–29.9 kg/m^2^) and obese (BMI ≥30 kg/m^2^) status. The median age was 76.0 years (IQR 70.0–82.0), and 41.7% of patients were women. In the overweight group (*n* = 4294 AF patients), patients on DOACs (*n* = 2427) were older, more frequently women and affected by arterial hypertension, and had a history of previous stroke or TIA compared to VKA. Conversely, patients on VKA (*n* = 1867) were more frequently affected by CAD, severe chronic kidney disease with eGFR <30 mL/min/1.73 m^2^ and HF. A significant difference in the management of rhythm and rate control strategies and antiplatelet use was found between the two groups.

**TABLE 1:** Characteristics of enrolled patients according to overweight/obesity status.

	*N*	Overall, *N* = 6534	Overweight *N* = 4294		*p* value *DOAC* vs. *VKA*	Obesity *N* = 2240		*p*‐value *DOAC* vs. *VKA*	*p*‐value *DOAC* vs *DOAC*	*p*‐value *VKA* vs. *VKA*
			VKA (*n*:1867)	DOAC (*n*: 2427)		VKA (*n*: 978)	DOAC (*n*: 1262)			
Age (years)^a^	6258	76.0 (70.0, 82.0)	76.0 (70.0, 81.0)	78.0 (72.0, 83.0)	<0.001	73.0 (66.0, 79.0)	76.0 (69.0, 81.0)	<0.001	<0.001	<0.001
Age ≥ 75 years	6258	3780 (57.9%)	1069 (57.3)	1617 (66.7)	<0.001	414 (42.3)	680 (54.0)	<0.001	<0.001	<0.001
Women		2726 (41.7%)	717 (38.4)	977 (40.3)	0.218	444 (45.4)	588 (46.6)	0.574	<0.001	<0.001
BMI^a^	6534	28.4 (26.6, 31.2)	27.1 (26.0, 28.4)	27.1 (26.0, 28.4)	0.575	32.7 (31.1, 35.2)	32.8 (31.2, 35.2)	0.567	<0.001	<0.001
Obesity degree	6534				‐			0.042	<0.001	<0.001
Obesity I		1651 (25.3%)	0.0	0.0		723 (73.9)	928 (73.5)			
Obesity II		413 (6.3%)	0.0	0.0		165 (16.9)	248 (19.7)			
Obesity III		176 (2.7%)	0.0	0.0		90 (9.2)	86 (6.8)			
Hypertension	6534	5478 (83.8%)	1477 (79.1%)	2038 (84.0)	<0.001	832 (85.1)	1131 (89.6)	0.001	<0.001	<0.001
Diabetes	6534	1610 (24.6%)	383 (20.5)	492 (20.3)	0.845	328 (33.5)	407 (32.3)	0.520	<0.001	<0.001
CAD	6534	1140 (17.5%)	409 (21.9)	365 (15.0)	<0.001	185 (18.9)	181 (14.3)	0.004	0.572	0.062
Anaemia	6534	1608 (24.6%)	486 (26.0)	604 (24.9)	0.393	226 (23.1)	292 (23.1)	0.987	0.240	0.087
eGFR^a^	6521	68.0 (52.0, 89.0)	64.0 (48.0, 81.0)	64.0 (50.0. 80.0)	0.062	82.0 (58.5. 105.0)	79.0 (60.0, 101.0)	0.925	<0.001	<0.001
eGFR <30 ml/min	6521	192 (2.9%)	114 (6.1)	30 (1.2)	<0.001	42 (4.3)	6 (0.5)	<0.001	0.026	0.043
Heart failure	6534	1487 (22.8%)	441 (23.6)	485 (20.0)	0.004	251 (25.7)	310 (24.6)	0.551	0.001	0.227
Smoking	6534	297 (4.5%)	95 (5.1)	92 (3.8)	0.039	50 (5.1)	60 (4.8)	0.697	0.162	0.978
Previous stroke/TIA	6534	964 (15%)	263 (14.1)	406 (16.7)	0.018	116 (11.9)	179 (14.2)	0.107	0.045	0.097
PAD	6534	393 (6.0%)	133 (7.1)	140 (5.8)	0.071	61 (6.2)	59 (4.7)	0.103	0.163	0.373
COPD/OSAS	6534	817 (12.5%)	207 (11.1)	251 (10.3)	0.433	150 (15.3)	209 (16.6)	0.434	<0.001	0.001
CHA_2_DS_2_‐VASc^a^	6528	4.0 (3.0, 5.0)	4.0 (2.0, 5.0)	4.0 (3.0, 5.0)	0.002	4.0 (2.0, 4.0)	4.0 (3.0, 5.0)	<0.001	0.119	0.675
HAS‐BLED^a^	6528	2.0 (2.0, 2.0)	2.0 (2.0, 3.0)	2.0 (2.0, 2.0)	0.092	2.0 (1.0, 2.0)	2.0 (2.0, 2.0)	<0.001	0.507	0.005
*Therapy*										
Anticoagulant type	6534				<0.001			<0.001	<0.001	‐
VKA		2845 (43.7%)	100.0	0.0		100.0	0.0			
Apixaban		1142 (17.5%)	0.0	717 (29.5)		0.0	425 (33.7)			
Rivaroxaban		1016 (15.6%)	0.0	696 (28.7)		0.0	320 (25.4)			
Edoxaban		621 (9.5%)	0.0	448 (18.5)		0.0	173 (13.7)			
Dabigatran		910 (13.9%)	0.0	566 (23.3)		0.0	344 (27.3)			
TTR	1867	67.0 (56.0, 76.0)	68.0 (57.0, 77.0)	‐	‐	65.0 (53.0, 75.0)	‐	‐	‐	0.001
TTR in range (≥60%)	1867	1271 (68%)	879 (71%)	‐	‐	392 (62%)	‐	‐	‐	<0.001
DOAC Reduced Dose	3689	1298 (35%)	‐	938 (39%)	‐	‐	360 (29%)	‐	<0.001	‐
Antiplatelet	6534	786 (12.0%)	286 (15.3)	243 (10.0)	<0.001	128 (13.1)	129 (10.2)	0.035	0.841	0.109
Class 1c AAD	6534	530 (8.1%)	118 (6.4)	249 (10.3)	<0.001	61 (6.3)	102 (8.1)	0.101	0.032	0.913
Amiodarone	6534	840 (12.9%)	273 (14.6)	282 (11.6)	0.004	138 (14.1)	147 (11.6)	0.083	0.979	0.712
Lipid lowering therapy	6534	2456 (37.6%)	690 (37.0)	885 (36.5)	0.740	366 (37.4)	515 (40.8)	0.104	0.010	0.807
RAASi	6534	3920 (60.0%)	1084 (58.1)	1432 (59.0)	0.535	613 (62.7)	791 (62.7)	1.000	0.030	0.017
Beta blockers	6534	3095 (47.4%)	1004 (53.8)	1008 (41.5)	<0.001	562 (57.5)	521 (41.3)	<0.001	0.884	0.060
Calcium channel blockers	6534	1558 (23.8%)	479 (25.7)	509 (21.0)	<0.001	271 (27.7)	299 (23.7)	0.030	0.058	0.238
Diuretics	6534	2662 (40.7%)	687 (36.8)	926 (38.2)	0.363	458 (46.8)	591 (46.8)	1.000	<0.001	<0.001
Digoxin	6534	516 (7.9%)	162 (8.7)	169 (7.0)	0.037	92 (9.4)	93 (7.4)	0.082	0.649	0.517
PPI	6534	2329 (35.6%)	704 (37.7)	815 (33.6)	0.005	362 (37.0)	448 (35.5)	0.459	0.244	0.717

Abbreviations: AAD, anti‐arrhythmic drugs; AF, Atrial Fibrillation; BMI, body mass index; CAD, coronary artery disease; COPD/OSAS, chronic obstructive pulmonary disease/obstructive sleep apnoea syndrome; DOAC, direct oral anticoagulants; eGFR, estimated glomerular filtration rate; PAD, peripheral artery disease; PPI, proton pump inhibitors; RAASi, renin‐angiotensin‐aldosterone inhibitors; TIA, transient ischaemic attack; TTR, Time in Therapeutic Range; VKA, vitamin K antagonist.

^a^ Median and interquartile range (IQR).

On the other hand, in the obesity groups (*n* = 2240 AF patients), patients on DOACs (*n* = 1262) were older, more frequently women and affected by arterial hypertension compared to VKA. AF patients on VKA (*n* = 978) were more frequently affected by CAD, severe chronic kidney disease with eGFR <30 mL/min/1.73 m^2^, and had a higher proportion of patients with a severe degree of obesity.

Significant differences in the management of rate control (i.e. calcium channel blockers and beta blockers) strategy and antiplatelet were found between two groups (Table 1).

Baseline characteristics of the 2240 obese patients, stratified by obesity class Obesity I (BMI 30.0–34.9 kg/m^2^, *n* = 1651), Obesity II (BMI 35.0–39.9 kg/m^2^, *n* = 413), and Obesity III (BMI ≥40.0 kg/m^2^, *n* = 176) and explorative comparisons are presented in Table S4.

### 3.3 Survival analysis

**FIGURE 1:**
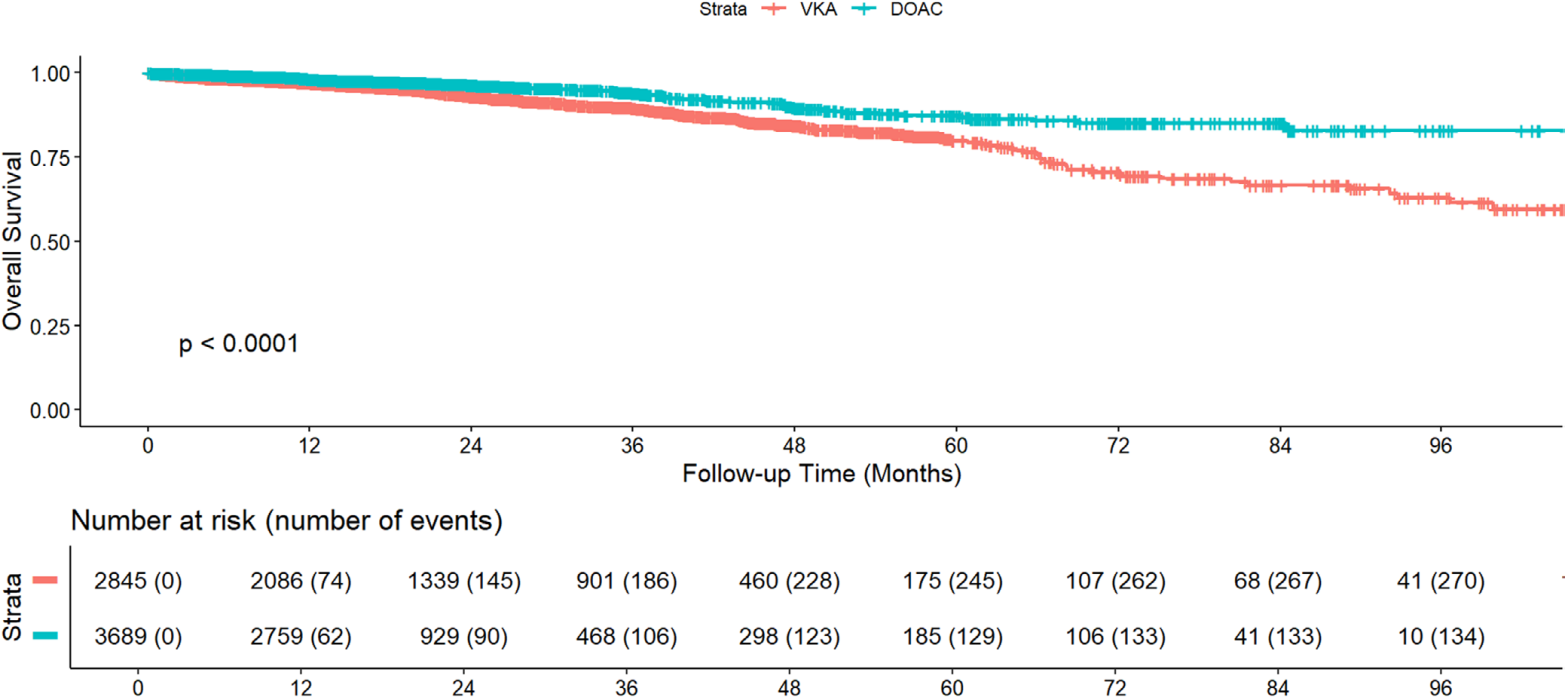
Kaplan–Meier survival curves by anticoagulant type.

During follow‐up, 408 all‐cause deaths, and 481 CVEs occurred. The Kaplan–Meier curve in Figure 1 illustrates overall survival in patients with BMI >25 kg/m^2^, stratified by type of oral anticoagulant (DOAC vs. VKA). Over a median follow‐up of 17.4 [IQR 12.0–30.6] months, patients treated with DOACs demonstrated significantly better survival compared to those on VKAs (log‐rank *p* < 0.0001).

The results of the univariable and multivariable Cox proportional hazards models for all‐cause mortality are summarized in Table 2. In the multivariable model, treatment with DOACs was independently associated with a significantly lower risk of all‐cause mortality compared to VKAs (HR 0.57, 95% CI 0.46–0.70, *p* < 0.001). This result was then confirmed by performing univariable Cox regression analysis on the PSM cohort (HR 0.49, 95% CI 0.38–0.62, *p* < 0.001).

**TABLE 2:** Univariable and multivariable cox regression models for predictors of all‐cause mortality in the overall population with body mass index ≥25 Kg/m^2^.

	Univariable			Multivariable		
	HR	95% CI	*p*‐value	HR	95% CI	*p*‐value
DOAC (vs VKA)	0.54	0.44, 0.67	<0.001	0.57	0.46, 0.70	<0.001
Obesity (vs *overweight*)	1.00	0.81, 1.22	0.962			
Age ≥75 years	3.09	2.42, 3.94	<0.001	1.91	1.45, 2.50	<0.001
Women	1.11	0.91, 1.34	0.311			
Hypertension	1.11	0.83, 1.47	0.482			
Diabetes	1.16	0.93, 1.44	0.189			
CAD	1.60	1.28, 2.01	<0.001	1.39	1.08, 1.78	0.011
Anaemia	2.18	1.78, 2.66	<0.001	1.41	1.14, 1.75	0.002
eGFR	0.97	0.97, 0.98	<0.001	0.99	0.98, 0.99	<0.001
Heart failure	1.91	1.56, 2.35	<0.001	1.32	1.05, 1.65	0.017
Smoking	0.77	0.44, 1.33	0.347			
Previous stroke/TIA	1.18	0.92, 1.52	0.191			
PAD	2.16	1.59, 2.95	<0.001	1.77	1.29, 2.43	<0.001
COPD/OSAS	2.05	1.61, 2.59	<0.001	1.58	1.23, 2.03	<0.001
Antiplatelet	1.24	0.93, 1.64	0.137			
Class 1c AAD	0.35	0.20, 0.62	<0.001	0.52	0.29, 0.92	0.026
Amiodarone	0.95	0.71, 1.27	0.726			
Lipid lowering therapy	0.75	0.61, 0.92	0.006	0.67	0.54, 0.85	<0.001
RAASi	0.66	0.55, 0.81	<0.001	0.67	0.55, 0.81	<0.001
Beta blockers	0.96	0.79, 1.17	0.683			
Calcium channel blockers	1.09	0.87, 1.35	0.456			
Diuretics	1.64	1.35, 1.99	<0.001	1.07	0.86, 1.33	0.532
Digoxin	1.07	0.77, 1.49	0.690			
PPI	1.12	0.91, 1.36	0.280			

Abbreviations: AAD, anti‐arrhythmic drugs; AF, atrial fibrillation; BMI, body mass index; CAD, coronary artery disease; CI, confidence interval; COPD/OSAS, chronic obstructive pulmonary disease/obstructive sleep apnoea syndrome; DOAC, direct oral anticoagulants; eGFR, estimated glomerular filtration rate; HR, hazard ratio; PAD, peripheral artery disease; PPI, proton pump inhibitors; RAASi, renin‐angiotensin‐aldosterone inhibitors; TIA, transient ischaemic attack; VKA, vitamin K antagonist.

Obesity status (vs overweight) was not associated with mortality (HR 1.00, 95% CI 0.81–1.22, *p* = 0.962). In contrast, age ≥75 years, CAD, anaemia, HF, PAD, COPD/OSAS and reduced eGFR were associated with mortality (Table 2). Among pharmacological treatments, the use of lipid‐lowering therapy, RAAS inhibitors, and Class 1c antiarrhythmics was associated with a lower risk of death (Table 2).

Similar results were observed in the multivariable model including clinically relevant covariates and an interaction term for anticoagulant type and BMI Class (Table S5), DOAC use was associated with a significantly lower risk of all‐cause mortality (HR 0.41, 95% CI 0.31–0.54, *p* < 0.001) compared with VKAs. Obesity, relative to overweight, was not independently associated with either outcome, and the interaction term did not reach statistical significance.

Table S6 reports the results of separate Cox regression analyses in overweight (Panel A) and obese (Panel B) patients. In both groups, DOAC therapy remained associated with a lower risk of all‐cause mortality (Overweight: HR 0.52, 95% CI 0.39–0.68; Obese: HR 0.69, 95% CI 0.48–0.98) after adjustment.

### 3.4 Predictors of CVEs

The overall incidence rate of CVEs was 3.69 per 100 person‐years (95% CI: 3.37–4.04). CIF curves are shown in Figure S2. Results of Fine‐Grey competing risk regression identifying predictors of CVEs are shown in Table 3. In the overall population, DOAC use was associated with a lower subdistribution hazard for CVEs compared to VKA, both in univariable (sHR 0.68, 95% CI 0.56–0.81, *p* < 0.001) and multivariable models (sHR 0.71, 95% CI 0.59–0.86, *p* < 0.001). This result was then confirmed by performing univariable Fine‐Grey analysis on the PSM cohort (sHR 0.63, 95% CI 0.51–0.78, *p* < 0.001).

**TABLE 3:** Univariable and multivariable fine–grey competing risk models for predictors of cardiovascular events in the overall population with body mass index ≥25 Kg/m^2^.

	Univariable			Multivariable		
	sHR	95% CI	*p*‐value	sHR	95% CI	*p*‐value
DOAC (vs VKA)	0.68	0.56, 0.81	<0.001	0.71	0.59, 0.86	<0.001
Obesity (vs *overweight*)	0.99	0.82, 1.20	0.952			
Age ≥75 years	2.63	2.12, 3.27	<0.001	1.66	1.30, 2.11	<0.001
Women	1.05	0.88, 1.26	0.569			
Hypertension	1.11	0.85, 1.44	0.450			
Diabetes	1.22	1.00, 1.49	0.054			
CAD	1.65	1.34, 2.02	<0.001	1.44	1.14, 1.83	0.003
Anaemia	1.96	1.62, 2.36	<0.001	1.34	1.10, 1.63	0.004
eGFR	0.98	0.97, 0.98	<0.001	0.99	0.98, 0.99	<0.001
Heart failure	1.80	1.49, 2.18	<0.001	1.29	1.05, 1.60	0.017
Smoking	0.97	0.61, 1.53	0.880			
Previous stroke/TIA	1.21	0.96, 1.52	0.106			
PAD	2.17	1.63, 2.89	<0.001	1.79	1.33, 2.40	<0.001
COPD/OSAS	1.91	1.53, 2.39	<0.001	1.52	1.20, 1.92	<0.001
Antiplatelet	1.43	1.12, 1.83	0.004	1.04	0.80, 1.36	0.761
Class 1c AAD	0.42	0.26, 0.67	<0.001	0.58	0.35, 0.94	0.028
Amiodarone	0.91	0.69, 1.20	0.507			
Lipid lowering therapy	0.82	0.68, 0.99	0.036	0.72	0.58, 0.88	0.002
RAASi	0.76	0.64, 0.91	0.003	0.76	0.63, 0.91	0.003
Beta blockers	0.87	0.73, 1.05	0.141			
Calcium channel blockers	1.05	0.86, 1.29	0.623			
Diuretics	1.48	1.24, 1.77	<0.001	0.99	0.81, 1.21	0.896
Digoxin	0.99	0.72, 1.35	0.943			
PPI	1.01	0.84, 1.22	0.906			

Abbreviations: AAD, anti‐arrhythmic drugs; AF, atrial fibrillation; BMI, body mass index; CAD, coronary artery disease; CI, confidence interval; COPD/OSAS, chronic obstructive pulmonary disease/obstructive sleep apnoea syndrome; DOAC, direct oral anticoagulants; eGFR, estimated glomerular filtration rate; PAD, peripheral artery disease; PPI, proton pump inhibitors; RAASi, renin‐angiotensin‐aldosterone inhibitors; sHR, Subdistribution Hazard Ratio; TIA, transient ischaemic attack; VKA, vitamin K antagonist.

Obesity was not associated with a difference in CVE risk compared to overweight (sHR 0.99, 95% CI 0.82–1.20, *p* = 0.952). Advanced age (≥75 years), CAD, anaemia, HF, PAD, and COPD/OSAS emerged as independent predictors of increased CVE risk. Conversely, lipid‐lowering therapy (sHR 0.72, 95% CI 0.58–0.88, *p* = 0.002), RAAS inhibitors (sHR 0.76, 95% CI 0.63–0.91, *p* = 0.003), and class 1c antiarrhythmics (sHR 0.58, 95% CI 0.35–0.94, *p* = 0.028) were associated with reduced risk.

Similar results were also observed in the second model, including only clinically relevant covariates and the interaction term between anticoagulant type and BMI Class (Table S5), DOAC use was associated with significantly lower risk of CVEs (sHR 0.55, 95% CI 0.43–0.71, *p* < 0.001) compared with VKAs, with no statistical significance of the interaction term.

In stratified Fine–Grey analyses (Table S7), DOAC use remained significantly associated with reduced risk of CVEs among overweight patients (sHR 0.67, 95% CI 0.53–0.86, *p* = 0.002), but this association did not reach statistical significance in the obese subgroup (sHR 0.80, 95% CI 0.58–1.09, *p* = 0.153). Among overweight patients, several clinical factors—including age ≥75 years, CAD, PAD, and COPD/OSAS, and reduced eGFR—were robust predictors of increased CVE risk. Similar risk factors were identified in the obese group, with significant contributions from HF, anaemia, PAD, and prior stroke/TIA. Notably, lipid‐lowering therapy and RAAS inhibitors were protective in overweight individuals, but not in those with obesity (Table S7).

### 3.5 Exploratory analyses

In the univariable Cox model with RCS, BMI as a continuous variable was not significantly associated with all‐cause mortality (overall effect *p* = 0.76; test for nonlinearity *p* = 0.70, Figure S3). The spline curve showed a relatively flat relationship between BMI and mortality, indicating no clear evidence of increased or reduced risk across the BMI spectrum, which is consistent with the findings of the other Cox models. Then, we performed an analysis stratifying patients according to TTR class (threshold 60%). TTR data were available for 1867 patients (65.6% of the cohort), of whom 1271 (68%) had TTR ≥60%. Table S8 reports the results of multivariable Cox regression for all‐cause mortality and Fine–Grey models for CVEs, stratified by TTR. In patients with TTR ≥60% (Panel A), DOAC use was associated with a lower risk of all‐cause mortality (HR 0.47, 95% CI 0.35–0.63, *p* < 0.001) and CVEs (sHR 0.64, 95% CI 0.49–0.83, p < 0.001) compared to VKA. Obesity was not significantly associated with outcomes, and the interaction term between DOAC and obesity was not statistically significant. Among patients with TTR <60% (Panel B), DOAC use remained strongly protective for all‐cause mortality (HR 0.32, 95% CI 0.23–0.44, p < 0.001) and CVEs (sHR 0.43, 95% CI 0.33–0.58, *p* < 0.001). The interaction between DOAC and obesity was statistically significant for all‐cause mortality, suggesting that in patients with suboptimal TTR, the mortality benefit of DOAC may be attenuated in obese patients. No significant interaction was observed for CVEs.

Third, although the low number of patients with severe obesity, we performed an exploratory analysis, to evaluate the use of DOAC, compared to warfarin use, in each subgroup of patients according to obesity severity. At univariable Cox analysis (Table S9), we observed a reduction in the all‐cause of death risk in overweight and obesity 1st degree severity patients treated with DOAC, without difference between DOAC and VKA use in patients with 2nd and 3rd degree of obesity (Table S9A). For CVEs, a univariable Fine‐Grey competing risk analysis showed a lower incidence of CVEs in overweight patients treated with DOAC, while no differences were detected across the higher obesity categories (Table S9B).

Fourth, we performed a subgroup analysis restricted to patients with body weight ≥100 kg. Overall, 708 patients (10.8% of the cohort) were in this group, accounting for 41 deaths and 51 CVEs. In univariable models, DOAC use was not significantly associated with all‐cause mortality (HR 0.88, 95% CI 0.47–1.65, *p* = 0.691) or with CVEs (sHR 0.95, 95% CI 0.54–1.67, *p* = 0.850).

**FIGURE 2:**
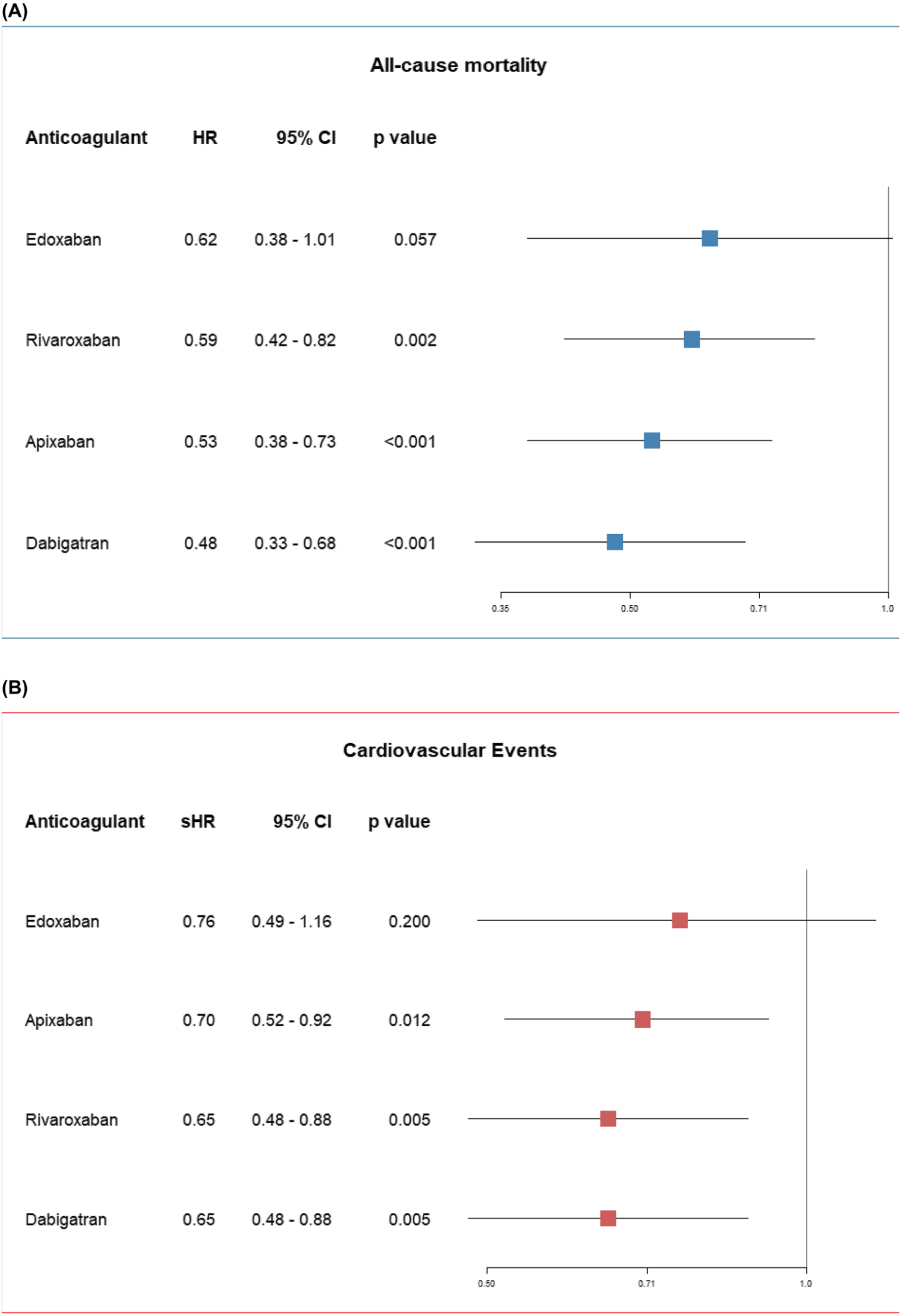
Forest plot showing the association of individual DOAC agents versus VKA with all‐cause mortality (A) and cardiovascular events (B).

Lastly, we examined the association of individual DOAC agents with outcomes, using VKA as the reference (Figure 2). For both all‐cause mortality (Panel A) and CVEs (Panel B), Apixaban, Rivaroxaban, and Dabigatran were associated with lower risk, whereas Edoxaban did not reach statistical significance.

## 4 DISCUSSION

In this large, real‐world cohort of AF patients enrolled in the START registry, we found a high prevalence of overweight obesity accounting for nearly two‐thirds of the total population, with diverse distribution across Italian regions. The distribution in our cohort reflects the Italian general population with a higher prevalence of overweight and obesity in southern countries of Italy^23^ and older adults.^23^ However, although we found a similar prevalence of overweight in AF patients (40.8%) compared to the general population with age ≥75 years (41.3%),^23^ we found a higher prevalence of obesity in our cohort (23.1%) compared to the overall population (13.4%) with a similar age observed in the literature.^23^ This finding confirms that patients with AF are affected by multiple cardio‐metabolic risk factors.

Despite this high prevalence and although obese patients exhibited a higher burden of cardiometabolic comorbidities, including hypertension, diabetes, and COPD/OSAS, compared to overweight patients, obesity was not associated with an increased risk of all‐cause mortality or CVEs.

In exploratory analyses, RCS modelling showed a relatively flat relationship between BMI and mortality, indicating no clear evidence of either increased or reduced risk across the BMI spectrum. At very high body weight, DOAC use was not associated with either outcome, although this may reflect limited statistical power due to the small number of events.

In contrast, we observed a significant effect of eGFR on both all‐cause mortality and CVE risk across different modelling strategies. This aspect has been explored in detail in a previous publication.^24^

Importantly, treatment with DOACs was associated with a lower risk of both all‐cause mortality and CVEs, with consistent effects observed in overweight patients. However, in the obese subgroup, the inverse association of DOACs with CVEs did not reach statistical significance, suggesting potential effect modification by body size or residual confounding.

A retrospective study from the ARISTOPHANES cohort^14^ performed on 88,461 AF patients with obesity (BMI ≥30 kg/m^2^) found a lower risk of ischemic stroke and systemic embolism in patients treated with apixaban and rivaroxaban compared to VKA. However, patients were followed for a short period (6.9 months) and no data were available on other clinical outcomes such as all‐cause mortality and CVEs and about concomitant comorbidities and therapies.^14^

A post‐hoc analysis of the ENSURE‐AF^15^ trial, which included 2199 AF patients undergoing cardioversion showed no significant differences between warfarin and DOAC in reducing a composite endpoint of stroke, systemic embolic event, myocardial infarction, cardiovascular death, and regarding safety, (composite of major and clinically relevant non‐major bleeding). However, the short follow‐up (2 months), the younger age (mean age 63 years) and the peculiar clinical setting as patients undergoing cardioversion for AF may explain the differences in the results.

A further study performed on 28,011 veterans with AF from the retrospective registry Veterans Health Administration Corporate Data Warehouse^17^ showed no difference in the effectiveness of DOAC and VKA, although DOACs were associated with a lower risk of bleeding compared to VKA. However, the cohort enrolled only patients with severe obesity (weight ≥120 kg or BMI >40 kg/m^2^) and the population was younger (about 63 years) compared to our AF cohort and it is made up almost exclusively of male veterans, underlining a potential selection bias.

Another retrospective analysis from 2 healthcare claims databases^16^ enrolling 3563 matched pairs of morbidly obese AF, defined according to International Classification of Diseases‐9 (ICD‐9), showed similar effectiveness of ischemic stroke and systemic embolism between rivaroxaban and warfarin with a lower risk of bleeding in the rivaroxaban group. Patients enrolled were younger than our cohort (mean age 63 years), were treated only with rivaroxaban instead of all DOACs and were followed only for a mean of 10.3 months. Furthermore, no data about all‐cause mortality and CVEs were available.

Additionally, a retrospective study performed^18^ on 29,135 AF patients followed for a mean of 44.4 months showed no difference in bleeding and ischemic stroke between oral anticoagulants but a higher mortality rate in patients underweight and with obesity degree I treated with DOACs. The cohort was similar to our population with a mean age of 76 years and a high thromboembolic risk (mean CHA_2_DS_2_‐VASc of 3.5). The higher mortality risk may be explained because of concomitant diseases: indeed, patients on DOACs, compared to VKA, had a greater proportion of the elderly and comorbidities, as also explained by Nakao et al.^18^

Finally, a recent metanalysis,^25^ including 434,320 obese patients with either AF or venous thromboembolism, found similar results to our study: indeed, they observed a reduction in the composite outcome (all‐cause mortality, stroke, systemic embolism, and myocardial infarction). However, the heterogeneity of the studies included and population characteristics, the inclusion of both patients with AF and venous thromboembolism, the different doses of anticoagulation by clinical indication and the inclusion of several retrospective studies represented limitations and potential sources of bias in the analysis.

In synthesis, controversial results of previous studies performed in AF patients with obesity may be caused by different inclusion criteria (i.e. different obesity degrees included), different clinical outcomes, length of follow‐up, heterogeneity of comorbidity and age and the design of the studies, often retrospective or post‐hoc analyses of clinical trials with very restricted inclusion criteria.

### 4.1 Strengths

Our study had several strengths. Firstly, the prospective design could identify obese patients with a specific tool as BMI instead of ICD code. Furthermore, patients were consecutively enrolled and observed during a long follow‐up evaluating CVEs and all‐cause mortality, instead of only ischemic stroke risk. In addition, the START registry was well characterized with several comorbidities' information collected at baseline compared to retrospective studies that often were subjected to limited clinical information and selection bias. Finally, our multicenter cohort reflects real‐world data on patients with AF, an elderly population with multiple comorbidities and high thromboembolic and cardiovascular risk and may be useful to estimate the effect of DOAC in this older and frail population.

### 4.2 Limitations

However, our study has several limitations. First, this was a multicenter study conducted in Italy and included predominantly Caucasian patients; therefore, these findings may be generalizable to Western countries with a similar prevalence of obesity; however, further studies are needed to assess their applicability to other populations, such as Asian populations, which have different metabolic phenotypes and obesity prevalence. Additionally, patient recruitment was not evenly distributed across Italian regions, which may affect the regional representativeness of the sample. In particular, some regions contributed only a small number of patients, making it difficult to draw reliable conclusions about regional obesity patterns. Moreover, given the registry‐based nature of the study, 60 centers were involved, many of which enrolled only a small number of patients. This prevented us from performing a stratified analysis by center or including the center variable in the PSM, thereby limiting our ability to adjust for inter‐center variability; however, the large number of participating centers may have helped dilute any potential prescription bias, thus enhancing the generalizability of our findings.

Second, BMI has inherent limitations as a measure of adiposity, as it does not differentiate between fat and lean mass or reflect fat distribution, which are more closely linked to CV risk. Nevertheless, BMI was routinary and widely used as a practical surrogate of adiposity in common clinical practice.^13^, ^20^, ^21^

Moreover, our cohort enrolled a low proportion of patients with severe obesity (i.e. second degree or major) so our results in this subgroup of patients were merely exploratory and not conclusive.

Third, we do not have information on the occurrence of each single component of the composite CVE; this limits a detailed analysis of the relevance of each single event type. Similarly, we do not have information on adherence and persistence, which could potentially impact the effectiveness of anticoagulant treatment.

Finally, as common in observational studies, we could not exclude potential unmeasured confounders that may influence the results, although the extensive multivariable adjustment performed in our study took into account several comorbidities and treatments involved in the risk of all‐cause mortality and CVEs.

## 5 CONCLUSIONS

In conclusion, this real‐world study performed on AF patients showed a high prevalence of overweight and obesity in this population. Furthermore, the treatment with DOACs was independently associated with a lower risk of all‐cause mortality and CVEs compared to VKAs after adjustment for multiple clinical covariates in both overweight and obese patients. These findings seem to support the use of DOACs as an effective and safe therapeutic option across a broad range of elevated BMI categories and highlight the importance of individualized risk assessment beyond BMI alone. However, further studies are needed to confirm our results, especially in patients with extreme BMI and severe obesity degrees.

## AUTHOR CONTRIBUTIONS

**Danilo Menichelli:** conceptualization, formal analysis, writing – original draft. **Gianluca Gazzaniga:** formal analysis, writing – original draft, visualization. **Emilia Antonucci:** data curation, methodology, investigation. **Gualtiero Palareti:** visualization, supervision, writing – review and editing. **Daniela Poli:** data curation, methodology, investigation. **Pasquale Pignatelli:** visualization, supervision, writing – review and editing. **Daniele Pastori:** conceptualization, formal analysis, visualization, supervision, writing – review and editing. All Authors read and approve the last version of the manuscript.

## CONFLICT OF INTEREST STATEMENT

The authors declare they have no conflict of interest.

## Supporting information


**Table S1:** Schoenfeld Residuals Test for the Proportional Hazards Assumption. (A) Multivariable Cox regression model including predictors significant at univariable analysis. (B) Multivariable Cox regression model including clinically relevant covariates and the interaction between anticoagulant type and BMI class.
**Table S2:** Patients’ characteristics before and after Propensity Score Matching.
**Table S3:** Propensity score matching balance diagnostics.
**Table S4:** Baseline characteristics of obese patients stratified according to obesity class.
**Table S5:** Multivariable Cox Regression for All‐Cause Mortality and Fine‐Grey Model for CVEs, including clinically relevant covariates and an interaction term between Anticoagulant Type and BMI Class.
**Table S6:** Univariable and multivariable cox regression models for predictors of all‐cause mortality in overweight (Panel A) and obese (Panel B) patients.
**Table S7:** Univariable and multivariable fine–grey competing risk models for predictors of cardiovascular events in overweight (Panel A) and obese (Panel B) patients.
**Table S8:** TTR‐stratified analysis of anticoagulant users; multivariable cox regression for all‐cause mortality and fine‐grey model for cardiovascular events (Panel A: TTR ≥60%; Panel B: TTR <60%).
**Table S9:** Univariable cox regression analysis of direct oral anticoagulant use (compared to warfarin) on all‐cause mortality (A) and Fine‐Grey analysis for cardiovascular events (B) according to obesity degree.
**Figure S1:** Geographic distribution of patients of START registry with BMI >25 across Italian regions.
**Figure S2:** Cumulative incidence function of CVE by anticoagulant type.
**Figure S3:** Association of Body Mass Index as a continuous variable with all‐cause mortality as modelled by restricted cubic splines regression analysis.

## Data Availability

The data that support the findings of this study are available from the corresponding author upon reasonable request.

## APPENDIX A

**List of participating centers and investigators who contributed to the START2 AF Registry:** Benilde Cosmi, Division of Angiology and Blood Coagulation, IRCCS Azienda Ospedaliero‐Universitaria S. Orsola‐Malpighi Bologna, Italy. Daniela Poli, Elena Lotti, Martina Berteotti, Rossella Marcucci SOD Malattie Aterotrombotiche, Azienda Ospedaliero Universitaria‐Careggi, Firenze, Italy. Walter Ageno, Giovanna Colombo, SSD Degenza Breve Internistica, Dipartimento di Medicina Interna, Ospedale di Circolo e Fondazione Macchi, ASST Sette Laghi, Varese, Italy. Doris Barcellona, UOC Medicina Interna, Centro Emocoagulopatie, AOU Cagliari, Cagliari. Giovanni Barillari, Alessandra Poz Ambulatorio Malattie Emorragiche e Trombotiche, Medicina Trasfusionale di Udine, Presidio Ospedaliero Universitario “Santa Maria della Misericordia”, Udine, Italy. Salvatore Bradamante, Centro Trasfusionale, Centro Emostasi e Trombosi, Ospedale SS Annunziata, Taranto, Italy. Eugenio Bucherini, Monica Vastola, Luca Bucherini SS Medicina Vascolare e Angiologia, AUSL Romagna – Ravenna, Italy. Paola Casasco, Centro Diagnosi Trombosi e Sorveglianza Terapia Anticoagulanti, Immunoematologia Trasfusionale, Ospedale SS Antonio e Margherita, Tortona (AL). Antonio Ciampa, Dipartimento Medicina di Laboratorio, Laboratorio Analisi, Ospedale San Giuseppe Moscati, Avellino, Italy. Antonio Chistolini, Alessandra Serrao, Dipartimento di Medicina Traslazionale e di Precisione Sapienza Università di Roma – Roma, Italy. Luciano Crippa Ambulatorio Emostasi e Trombosi, UO Patologie Trombotiche Ospedale San Raffaele, Milano, Italy. Raimondo De Cristofaro, Erica De Candia, UOSD Malattie Emorragiche e Trombotiche, Dipartimento Diagnostica per Immagini, Radioterapia Oncologica ed. Ematologia, Fondazione Policlinico Universitario Agostino Gemelli IRCCS, Roma, Italy. Valeria De Micheli, Centro Emostasi e Trombosi, Medicina Generale, ASST Lecco, Lecco, Italy. Igor Diemberger, Giuseppe Boriani, Dipartimento Cardiotoraco Vascolare, IRCCS Azienda Ospedaliero‐Universitaria S. Orsola‐Malpighi Bologna, Italy. Marcello Di Nisio, Ettore Porreca Dipartimento di Medicina e scienze dell'invecchiamento, SSD Medicina Interna University “G.D'Annunzio” di Chieti–Pescara, Italy. Anna Falanga, Teresa Lerede, Università Bicocca Milano, Dept. Medicine and Surgery, Monza and Divisione di Immunoematologia e Medicina Trasfusionale and Centro Emostasi e Trombosi, ASST Papa Giovanni XXIII – Bergamo, Italy. Elvira Grandone, Donatella Colaizzo, Centro Trombosi, Casa del Sollievo e della Sofferenza, S.Giovanni Rotondo (Foggia), Italy. Antonio Insana, SC Laboratorio Analisi Chimico‐Cliniche e Microbiologia. A.O. Ordine Mauriziano, Torino, Italy. Nicola Lucio Liberato, UO Medicina Interna, Ambulatorio Terapia Anticoagulante Orale, AO Provincia di Pavia, Pavia, Italy. Domenico Lione, UOC di Patologia Clinica, Ospedale A Perrino, Brindisi, Italy. Rosa Maria Lombardi, Centro Emostasi e Trombosi, UO Medicina Interna, Azienda Ospedaliero‐Universitaria di Parma, Italy. Giacomo Lucarelli, Centro Trombosi, Ospedale Regionale F. Miulli, Acquaviva delle Fonti (Bari), Italy. Giuseppe Malcangi, Centro Emofilia e Trombosi, Policlinico di Bari, Bari, Italy. Catello Mangione, Trasfusionale, Ospedale di Galatina, Galatina (LE), Italy. Giuliana Martini Centro Emostasi, Spedali Civili di Brescia, Brescia, Italy. Marco Marzolo, UOS Angiologia Medica, Ospedale di Rovigo, Italy. Giovanni Nante, Dipartimento di Medicina, Azienda Ospedaliero‐Universitaria di Padova, Padova, Italy. Vincenzo Oriana, Centro Emofilia—Presidio Ospedaliero Morelli, Reggio Calabria. Carmelo Paparo, Laboratorio Analisi, Ospedale Maggiore Chieri, Torino, Italy. Paolo Pedico, UO di Medicina Trasfusionale, Presidio Ospedaliero Dimiccoli, Barletta (Bari), Italy. Simona Pedrini, Servizio di Laboratorio, Istituto Ospedaliero Fondazione Poliambulanza, Brescia, Italy. Vittorio Pengo, Dipartimento di Scienze Cardio‐Toraco‐Vascolari, AOU Padova, Padova, Italy. Antonietta Piana, Francesco Cibecchini, Centro Prevenzione e Cura Malattie Tromboemboliche e Centro TAO, I.R.C.C.S. Azienda Ospedaliera Universitaria San Martino‐IST, Genova, Italy. Simona Pezzella, UO Medicina Trasfusionale, Centro TAO, PO Battipaglia, Battipaglia, (Salerno), Italy. Pasquale Pignatelli, Daniele Pastori, Centro Trombosi, Dipartimento SCIAC, Sapienza Università di Roma, Italy. Vincenza Rossi, Centro Emostasi e Trombosi, UOC Ematologia, Azienda Ospedaliera di Cosenza, Italy. Lucia Ruocco, Paolo Chiarugi, UO di Analisi Chimico Cliniche, Azienda Ospedaliero Universitaria Pisana, Pisa, Italy. Serena Rupoli, Clinica di Ematologia, AOU Ospedali Riuniti di Ancona, Ancona, Italy. Domizio Serra, Servizio Analisi, Ospedale Evangelico Internazionale, Sede di Castelletto, Genova, Italy. Carmine Spataro, Margherita Reduzzi, Chiara Ambaglio SIMT ASST Bergamo Ovest – Ospedale di Treviglio, pavia Italy. Sophie Testa, Oriana Paoletti, Centro Emostasi e Trombosi, UUOO Laboratorio Analisi chimico‐cliniche e microbiologiche, ASST Cremona, Cremona, Italy. Andrea Toma, UOC di Patologia Clinica, Ambulatorio Terapia Anticoagulante Orale, O.C. “L. Cazzavillan” Arzignano, (Vicenza), Italy. Vincenzo Toschi, Dipartimento di Medicina Trasfusionale e di Ematologia, ASST Santi Paolo e Carlo, Milano Italy. Rita Carlotta Santoro Centro Emostasi e Trombosi, UO Emofilia e Patologie della Coagulazione, Dipartimento di Ematologia, Oncologia e Medicina Trasfusionale, Azienda Ospedaliera “Pugliese Ciaccio”, Catanzaro, Italy. Claudio Vasselli, Laboratorio Patologia Clinica, Policlinico Casilino, Roma, Roma Italy. Maddalena Loredana Zighetti, Marco Cattaneo, Gianmarco Podda U.O. Medicina II, Azienda Ospedaliera San Paolo di Milano, ASST Santi Paolo e Carlo, Milano, Italy.
