# Supplementary material for: Effectiveness of direct oral anticoagulants for all‐cause mortality and cardiovascular events in overweight and obese patients with atrial fibrillation: Insight from the nationwide START registry
Source: Eur J Clin Invest. 2025 Nov 13;56(1):e70148. doi: 10.1111/eci.70148 (PMC12820908; doi:10.1111/eci.70148)
Supplement: Supplementary file 1 — Table S1: Schoenfeld Residuals Test for the Proportional Hazards Assumption. (A) Multivariable Cox regression model including predictors significant at univariable analysis. (B) Multivariable Cox regression model including clinically relevant covariates and the interaction between anticoagulant type and BMI class. Table S2: Patients’ characteristics before and after Propensity Score Matching. Table S3: Propensity score matching balance diagnostics. Table S4: Baseline characteristics of obese patients stratified according to obesity class. Table S5: Multivariable Cox Regression for All‐Cause Mortality and Fine‐Grey Model for CVEs, including clinically relevant covariates and an interaction term between Anticoagulant Type and BMI Class. Table S6: Univariable and multivariable cox regression models for predictors of all‐cause mortality in overweight (Panel A) and obese (Panel B) patients. Table S7: Univariable and multivariable fine–grey competing risk models for predictors of cardiovascular events in overweight (Panel A) and obese (Panel B) patients. Table S8: TTR‐stratified analysis of anticoagulant users; multivariable cox regression for all‐cause mortality and fine‐grey model for cardiovascular events (Panel A: TTR ≥60%; Panel B: TTR <60%). Table S9: Univariable cox regression analysis of direct oral anticoagulant use (compared to warfarin) on all‐cause mortality (A) and Fine‐Grey analysis for cardiovascular events (B) according to obesity degree. Figure S1: Geographic distribution of patients of START registry with BMI >25 across Italian regions. Figure S2: Cumulative incidence function of CVE by anticoagulant type. Figure S3: Association of Body Mass Index as a continuous variable with all‐cause mortality as modelled by restricted cubic splines regression analysis. [file ECI-56-e70148-s001.docx]

**Effectiveness of direct oral anticoagulants for all-cause mortality and cardiovascular events in overweight and obese patients with atrial fibrillation: insight from the nationwide START registry**

**Supplementary Material**

[**Supplementary Table 1. Schoenfeld Residuals Test for the Proportional Hazards Assumption. Panel A: Multivariable Cox regression model including predictors significant at univariable analysis. Panel B: Multivariable Cox regression model including clinically relevant covariates and the interaction between anticoagulant type and BMI class. 2**](#_Toc210563784)

[**Supplementary Table 2. Patients characteristics before and after Propensity Score Matching 4**](#_Toc210563785)

[**Supplementary Table 3. Propensity Score Mathing Balance Diagnostics 5**](#_Toc210563786)

[**Supplementary Table 4. Baseline characteristics of obese patients stratified according to obesity class. 7**](#_Toc210563787)

[**Supplementary Table 5. Multivariable Cox Regression for All-Cause Mortality and Fine-Gray Model for CVEs, including clinically relevant covariates and an interaction term between Anticoagulant Type and BMI Class 9**](#_Toc210563788)

[**Supplementary Table 6. Univariable and multivariable Cox Regression Models for Predictors of All-Cause Mortality in Overweight (Panel A) and Obese (Panel B) Patients 10**](#_Toc210563789)

[**Supplementary Table 7. Univariable and multivariable Fine–Gray Competing Risk Models for Predictors of Cardiovascular Events in Overweight (Panel A) and Obese (Panel B) Patients 12**](#_Toc210563790)

[**Supplementary Table 8. TTR-Stratified Analysis of Anticoagulant Users; Multivariable Cox Regression for All-Cause Mortality and Fine-Gray Model for Cardiovascular Events (Panel A: TTR ≥60%; Panel B: TTR <60%). 14**](#_Toc210563791)

[**Supplementary Table 9. Univariable Cox regression analysis of direct oral anticoagulant use (compared to warfarin) on all-cause mortality (Panel A) and Fine-Gray analysis for cardiovascular events (Panel B) according to obesity degree. 17**](#_Toc210563792)

[**Supplementary Figure 1. Geographic distribution of patients of START registry with BMI >25 across Italian regions. 19**](#_Toc210563793)

[**Supplementary Figure 2. Cumulative incidence function of CVE by anticoagulant type 20**](#_Toc210563794)

[**Supplementary Figure 3. Association of Body Mass Index as continuous variable with all-cause mortality as modelled by restricted cubic splines regression analysis. 21**](#_Toc210563795)

## Supplementary Table 1. Schoenfeld Residuals Test for the Proportional Hazards Assumption. Panel A: Multivariable Cox regression model including predictors significant at univariable analysis. Panel B: Multivariable Cox regression model including clinically relevant covariates and the interaction between anticoagulant type and BMI class.

**Panel A**

| **Variable** | **Chi-sq** | **df** | **p** |
| --- | --- | --- | --- |
| **DOAC (vs VKA)** | 1.72 | 1 | 0.189 |
| **Age ≥75 years** | 2.11 | 1 | 0.147 |
| **CAD** | 0.243 | 1 | 0.622 |
| **Anemia** | 0.000012 | 1 | 0.997 |
| **eGFR** | 1.85 | 1 | 0.174 |
| **HF** | 1.52 | 1 | 0.217 |
| **PAD** | 0.332 | 1 | 0.564 |
| **COPD/OSAS** | 2.97 | 1 | 0.085 |
| **Class 1c AAD** | 0.043 | 1 | 0.836 |
| **Lipid lowering therapy** | 0.399 | 1 | 0.528 |
| **RAASi** | 0.001 | 1 | 0.976 |
| **Diuretics** | 3.24 | 1 | 0.072 |
| **GLOBAL** | 15.2 | 12 | 0.231 |

**Panel B**

| **Variable** | **Chisq** | **df** | **p** |
| --- | --- | --- | --- |
| **Anticoagulant Type** | 1.64 | 1 | 0.201 |
| **BMI Class** | 0.0972 | 1 | 0.755 |
| **Age (years)** | 0.226 | 1 | 0.635 |
| **Female** | 0.0283 | 1 | 0.866 |
| **Hypertension** | 2.21 | 1 | 0.137 |
| **Diabetes** | 0.000919 | 1 | 0.976 |
| **CAD** | 0.562 | 1 | 0.453 |
| **PAF** | 1.07 | 1 | 0.301 |
| **Anaemia** | 0.000281 | 1 | 0.987 |
| **eGFR** | 1.72 | 1 | 0.190 |
| **Heart Failure** | 2.48 | 1 | 0.115 |
| **PAD** | 0.204 | 1 | 0.652 |
| **COPD/OSAS** | 3.08 | 1 | 0.079 |
| **Interaction term: DOAC * Obesity** | 0.746 | 1 | 0.388 |
| **GLOBAL** | 14.3 | 14 | 0.425 |

*Abbreviations: AAD, antiarrhythmic drug; BMI, body mass index; CAD, coronary artery disease; COPD/OSAS, chronic obstructive pulmonary disease/obstructive sleep apnea syndrome; DOAC, direct oral anticoagulant; eGFR, estimated glomerular filtration rate; HF, heart failure; PAF, paroxysmal atrial fibrillation; PAD, peripheral artery disease; RAASi, renin–angiotensin–aldosterone system inhibitor; VKA, vitamin K antagonist.*

## Supplementary Table 2. Patients characteristics before and after Propensity Score Matching

| **Characteristic** | **Before Matching** | | | **After Matching** | | |
| --- | --- | --- | --- | --- | --- | --- |
|  | **VKA** N = 2,841 | **DOAC** N = 3,677 | **SMD** | **VKA** N = 2,514 | **DOAC** N = 2,514 | **SMD** |
| Heart failure | 691 (24%) | 790 (21%) | -0.0691 | 580 (23%) | 570 (23%) | -0.0097 |
| Female | 1,158 (41%) | 1,557 (42%) | 0.0321 | 1,056 (42%) | 1,065 (42%) | 0.0072 |
| Age (years) | 75.00 (68.00, 81.00) | 77.00 (71.00, 83.00) | 0.3363 | 76.00 (70.00, 81.00) | 76.00 (70.00, 81.00) | 0.0483 |
| BMI | 28.40 (26.60, 31.20) | 28.40 (26.60, 31.20) | -0.0139 | 28.40 (26.60, 31.20) | 28.50 (26.60, 31.30) | -0.0017 |
| Hypertension | 2,306 (81%) | 3,158 (86%) | 0.1355 | 2,091 (83%) | 2,099 (83%) | 0.0091 |
| Diabetes | 710 (25%) | 897 (24%) | -0.0139 | 623 (25%) | 631 (25%) | 0.0074 |
| CAD | 593 (21%) | 542 (15%) | -0.173 | 459 (18%) | 442 (18%) | -0.0191 |
| Anaemia | 712 (25%) | 892 (24%) | -0.0187 | 603 (24%) | 606 (24%) | 0.0028 |
| eGFR | 68.00 (51.00, 89.00) | 68.00 (53.00, 88.00) | 0.0314 | 69.00 (51.00, 89.00) | 68.00 (54.00, 88.00) | 0.0107 |
| Previous stroke/TIA | 379 (13%) | 583 (16%) | 0.0689 | 355 (14%) | 354 (14%) | -0.0011 |
| COPD/OSAS | 357 (13%) | 458 (12%) | -0.0033 | 317 (13%) | 322 (13%) | 0.006 |
| Smoking | 143 (5.0%) | 152 (4.1%) | -0.0452 | 119 (4.7%) | 100 (4.0%) | -0.038 |
| Antiplatelet | 414 (15%) | 371 (10%) | -0.1488 | 315 (13%) | 298 (12%) | -0.0225 |
| Class 1c AAD | 179 (6.3%) | 349 (9.5%) | 0.1089 | 177 (7.0%) | 182 (7.2%) | 0.0068 |
| Amiodarone | 411 (14%) | 429 (12%) | -0.0872 | 321 (13%) | 318 (13%) | -0.0037 |
| Beta blockers | 1,564 (55%) | 1,527 (42%) | -0.2744 | 1,302 (52%) | 1,247 (50%) | -0.0444 |

*AAD: anti-arrhythmic drugs; AF: Atrial Fibrillation; BMI: body mass index; CAD: coronary artery disease, COPD/OSAS: chronic obstructive pulmonary disease/obstructive sleep apnoea syndrome; DOAC: direct oral anticoagulants; eGFR: estimated glomerular filtration rate; SMD: Standardized Mean Difference; TIA: transient ischaemic attack; VKA: vitamin K antagonist.*

## Supplementary Table 3. Propensity Score Mathing Balance Diagnostics

| **Summary of Balance for All Data** | | | | | | |  |
| --- | --- | --- | --- | --- | --- | --- | --- |
| **Variable** | **Means Treated** | **Means Control** | **Std. Mean Diff.** | **Var. Ratio** | **eCDF Mean** | **eCDF Max** |  |
| distance | 0.5989 | 0.5192 | 0.6303 | 0.7759 | 0.1603 | 0.2386 |  |
| Heart failure | 0.2148 | 0.2432 | -0.0691 | . | 0.0284 | 0.0284 |  |
| Female | 0.4234 | 0.4076 | 0.0321 | . | 0.0158 | 0.0158 |  |
| Age (years) | 76.5632 | 73.6325 | 0.3363 | 0.8271 | 0.0465 | 0.1282 |  |
| BMI | 29.4629 | 29.5189 | -0.0139 | 0.9361 | 0.0037 | 0.0134 |  |
| Hypertension | 0.8589 | 0.8117 | 0.1355 | . | 0.0472 | 0.0472 |  |
| Diabetes | 0.2439 | 0.2499 | -0.0139 | . | 0.006 | 0.006 |  |
| CAD | 0.1474 | 0.2087 | -0.173 | . | 0.0613 | 0.0613 |  |
| Anaemia | 0.2426 | 0.2506 | -0.0187 | . | 0.008 | 0.008 |  |
| eGFR | 72.9045 | 72.037 | 0.0314 | 0.7753 | 0.0144 | 0.0606 |  |
| Previous stroke/TIA | 0.1586 | 0.1334 | 0.0689 | . | 0.0251 | 0.0251 |  |
| COPD/OSAS | 0.1246 | 0.1257 | -0.0033 | . | 0.0011 | 0.0011 |  |
| Smoking | 0.0413 | 0.0503 | -0.0452 | . | 0.009 | 0.009 |  |
| Antiplatelet | 0.1009 | 0.1457 | -0.1488 | . | 0.0448 | 0.0448 |  |
| Class 1c AAD | 0.0949 | 0.063 | 0.1089 | . | 0.0319 | 0.0319 |  |
| Amiodarone | 0.1167 | 0.1447 | -0.0872 | . | 0.028 | 0.028 |  |
| Beta blockers | 0.4153 | 0.5505 | -0.2744 | . | 0.1352 | 0.1352 |  |
|  |  |  |  |  |  |  |  |
| **Summary of Balance for Matched Data** | | | | | | | |
| **Variable** | **Means Treated** | **Means Control** | **Std. Mean Diff.** | **Var. Ratio** | **eCDF Mean** | **eCDF Max** | **Std. Pair Dist.** |
| distance | 0.5598 | 0.5484 | 0.0902 | 1.0543 | 0.0262 | 0.0481 | 0.0904 |
| Heart failure | 0.2267 | 0.2307 | -0.0097 | . | 0.004 | 0.004 | 0.8678 |
| Female | 0.4236 | 0.42 | 0.0072 | . | 0.0036 | 0.0036 | 0.9942 |
| Age (years) | 75.2669 | 74.8461 | 0.0483 | 1.0038 | 0.0069 | 0.0243 | 0.9512 |
| BMI | 29.472 | 29.4788 | -0.0017 | 0.9159 | 0.0059 | 0.0163 | 1.0322 |
| Hypertension | 0.8349 | 0.8317 | 0.0091 | . | 0.0032 | 0.0032 | 0.7746 |
| Diabetes | 0.251 | 0.2478 | 0.0074 | . | 0.0032 | 0.0032 | 0.841 |
| CAD | 0.1758 | 0.1826 | -0.0191 | . | 0.0068 | 0.0068 | 0.7821 |
| Anaemia | 0.2411 | 0.2399 | 0.0028 | . | 0.0012 | 0.0012 | 0.8677 |
| eGFR | 72.5712 | 72.2749 | 0.0107 | 0.7342 | 0.0138 | 0.0533 | 1.0985 |
| Previous stroke/TIA | 0.1408 | 0.1412 | -0.0011 | . | 0.0004 | 0.0004 | 0.6589 |
| COPD/OSAS | 0.1281 | 0.1261 | 0.006 | . | 0.002 | 0.002 | 0.6541 |
| Smoking | 0.0398 | 0.0473 | -0.038 | . | 0.0076 | 0.0076 | 0.4256 |
| Antiplatelet | 0.1185 | 0.1253 | -0.0225 | . | 0.0068 | 0.0068 | 0.6907 |
| Class 1c AAD | 0.0724 | 0.0704 | 0.0068 | . | 0.002 | 0.002 | 0.4384 |
| Amiodarone | 0.1265 | 0.1277 | -0.0037 | . | 0.0012 | 0.0012 | 0.6926 |
| Beta blockers | 0.496 | 0.5179 | -0.0444 | . | 0.0219 | 0.0219 | 0.8193 |
|  |  |  |  |  |  |  |  |
| **Sample Sizes** | | |  |  |  |  |  |
|  | **Control** | **Treated** |  |  |  |  |  |
| All | 2841 | 3677 |  |  |  |  |  |
| Matched | 2514 | 2514 |  |  |  |  |  |
| Unmatched | 327 | 1163 |  |  |  |  |  |
| Diascarded | 0 | 0 |  |  |  |  |  |

## Supplementary Table 4. Baseline characteristics of obese patients stratified according to obesity class.

|  | N | Overall, N = 2,240 | Obesity I, N = 1,651 | Obesity II, N = 413 | Obesity III, N = 176 | p-value |
| --- | --- | --- | --- | --- | --- | --- |
| Age (years)* | 2,237 | 74.0 (68.0, 80.0) | 75.0 (69.0, 81.0) | 73.0 (66.0, 79.0) | 70.0 (63.8, 76.0) | <0.001 |
| Age ≥75 years | 2,237 | 1,094 (49%) | 871 (53%) | 171 (41%) | 52 (30%) | <0.001 |
| Women | 2,240 | 1,032 (46%) | 723 (44%) | 206 (50%) | 103 (59%) | <0.001 |
| BMI* | 2,240 | 32.8 (31.2, 35.2) | 31.7 (30.9, 33.1) | 36.6 (35.6, 37.8) | 42.7 (41.0, 45.8) | <0.001 |
| Hypertension | 2,240 | 1,963 (88%) | 1,435 (87%) | 366 (89%) | 162 (92%) | 0.116 |
| Diabetes | 2,240 | 735 (33%) | 482 (29%) | 178 (43%) | 75 (43%) | <0.001 |
| CAD | 2,240 | 366 (16%) | 285 (17%) | 58 (14%) | 23 (13%) | 0.135 |
| Anaemia | 2,240 | 518 (23%) | 383 (23%) | 98 (24%) | 37 (21%) | 0.768 |
| eGFR* | 2,235 | 80.0 (59.0, 103.0) | 76.0 (57.0, 98.0) | 90.0 (67.0, 113.0) | 107.0 (80.8, 141.3) | <0.001 |
| eGFR <30 ml/min | 2,235 | 48 (2.1%) | 38 (2.3%) | 7 (1.7%) | 3 (1.7%) | 0.796 |
| Heart failure | 2,240 | 561 (25%) | 414 (25%) | 108 (26%) | 39 (22%) | 0.591 |
| Smoking | 2,240 | 110 (4.9%) | 67 (4.1%) | 28 (6.8%) | 15 (8.5%) | 0.005 |
| Previous stroke/TIA | 2,240 | 295 (13%) | 240 (15%) | 41 (9.9%) | 14 (8.0%) | 0.005 |
| PAD | 2,240 | 120 (5.4%) | 93 (5.6%) | 23 (5.6%) | 4 (2.3%) | 0.166 |
| COPD/OSAS | 2,240 | 359 (16%) | 231 (14%) | 82 (20%) | 46 (26%) | <0.001 |
| CHA_2_DS_2_-VASc* | 2,237 | 4.0 (3.0, 5.0) | 4.0 (3.0, 5.0) | 4.0 (3.0, 5.0) | 3.0 (2.0, 4.0) | 0.073 |
| HAS-BLED* | 2,237 | 2.0 (2.0, 2.0) | 2.0 (2.0, 2.0) | 2.0 (2.0, 2.0) | 2.0 (1.0, 2.0) | <0.001 |
|  | Therapy | | | | | |
| Anticoagulant type | 2,240 |  |  |  |  | 0.244 |
| DOAC |  | 1,262 (56%) | 928 (56%) | 248 (60%) | 86 (49%) | 0.042 |
| Apixaban |  | 425 (19%) | 318 (19%) | 74 (18%) | 33 (19%) |  |
| Rivaroxaban |  | 320 (14%) | 231 (14%) | 67 (16%) | 22 (13%) |  |
| Edoxaban |  | 173 (7.7%) | 127 (7.7%) | 33 (8.0%) | 13 (7.4%) |  |
| Dabigatran |  | 344 (15%) | 252 (15%) | 74 (18%) | 18 (10%) |  |
| Antiplatelet | 2,240 | 257 (11%) | 201 (12%) | 41 (9.9%) | 15 (8.5%) | 0.194 |
| Class 1c AAD | 2,240 | 163 (7.3%) | 120 (7.3%) | 30 (7.3%) | 13 (7.4%) | 0.998 |
| Amiodarone | 2,240 | 285 (13%) | 206 (12%) | 52 (13%) | 27 (15%) | 0.554 |
| Lipid lowering therapy | 2,240 | 881 (39%) | 667 (40%) | 159 (38%) | 55 (31%) | 0.057 |
| RAASi | 2,240 | 1,404 (63%) | 1,027 (62%) | 271 (66%) | 106 (60%) | 0.344 |
| Beta blockers | 2,240 | 1,083 (48%) | 805 (49%) | 193 (47%) | 85 (48%) | 0.762 |
| Calcium channel blockers | 2,240 | 570 (25%) | 429 (26%) | 103 (25%) | 38 (22%) | 0.430 |
| Diuretics | 2,240 | 1,049 (47%) | 734 (44%) | 225 (54%) | 90 (51%) | <0.001 |
| Digoxin | 2,240 | 185 (8.3%) | 129 (7.8%) | 46 (11%) | 10 (5.7%) | 0.039 |
| PPI | 2,240 | 810 (36%) | 604 (37%) | 147 (36%) | 59 (34%) | 0.699 |

*AAD: anti-arrhythmic drugs; AF: Atrial Fibrillation; BMI: body mass index; CAD: coronary artery disease, COPD/OSAS: chronic obstructive pulmonary disease/obstructive sleep apnoea syndrome; DOAC: direct oral anticoagulants; eGFR: estimated glomerular filtration rate; PAD: peripheral artery disease; PPI: proton pump inhibitors; RAASi: renin-angiotensin-aldosterone inhibitors; TIA: transient ischaemic attack; VKA: vitamin K antagonist. *median and interquartile range (IQR)*

## Supplementary Table 5. Multivariable Cox Regression for All-Cause Mortality and Fine-Gray Model for CVEs, including clinically relevant covariates and an interaction term between Anticoagulant Type and BMI Class

| **Variable** | **All-cause mortality** | | | **CVEs** | | |
| --- | --- | --- | --- | --- | --- | --- |
|  | **HR** | **95% CI** | **p-value** | **sHR** | **95% CI** | **p-value** |
| **DOAC (vs VKA)** | 0.41 | 0.31, 0.54 | <0.001 | 0.55 | 0.43, 0.71 | <0.001 |
| **BMI Class: Obesity (Vs Overweight)** | 1.20 | 0.92, 1.57 | 0.178 | 1.20 | 0.94, 1.53 | 0.130 |
| **Interaction term: DOAC * Obesity*** | 1.45 | 0.94, 2.24 | 0.091 | 1.29 | 0.87, 1.90 | 0.200 |
| **Age (years)** | 1.08 | 1.07, 1.10 | <0.001 | 1.07 | 1.06, 1.09 | <0.001 |
| **Women** | 0.91 | 0.74, 1.12 | 0.379 | 0.88 | 0.73, 1.06 | 0.170 |
| **Hypertension** | 0.82 | 0.61, 1.09 | 0.166 | 0.82 | 0.64, 1.06 | 0.130 |
| **Diabetes** | 1.07 | 0.85, 1.35 | 0.558 | 1.15 | 0.93, 1.41 | 0.210 |
| **PAF** | 0.87 | 0.69, 1.09 | 0.235 | 0.99 | 0.81, 1.21 | 0.940 |
| **CAD** | 1.18 | 0.93, 1.50 | 0.179 | 1.26 | 1.01, 1.56 | 0.039 |
| **Anaemia** | 1.44 | 1.16, 1.78 | <0.001 | 1.32 | 1.08, 1.61 | 0.006 |
| **eGFR** | 0.99 | 0.99, 1.00 | 0.002 | 0.99 | 0.99, 1.00 | 0.003 |
| **Heart Failure** | 1.30 | 1.04, 1.61 | 0.021 | 1.27 | 1.03, 1.56 | 0.026 |
| **PAD** | 1.71 | 1.25, 2.35 | <0.001 | 1.71 | 1.28, 2.28 | <0.001 |
| **COPD/OSAS** | 1.50 | 1.17, 1.93 | 0.001 | 1.43 | 1.12, 1.83 | 0.004 |

**The reported interaction term quantifies the relative modification of DOAC’s effect in obese patients compared to non-obese. The real HR for DOAC in obese patients will be obtained by multiplying the main DOAC effect by the interaction term.*

*BMI: body mass index; CAD: coronary artery disease, COPD/OSAS: chronic obstructive pulmonary disease/obstructive sleep apnoea syndrome; DOAC: direct oral anticoagulants; eGFR: estimated glomerular filtration rate; PAF: Paroxysmal Atrial Fibrillation; PAD: peripheral artery disease; PPI: proton pump inhibitors; RAASi: renin-angiotensin-aldosterone inhibitors; TIA: transient ischaemic attack; VKA: vitamin K antagonist.*

## Supplementary Table 6. Univariable and multivariable Cox Regression Models for Predictors of All-Cause Mortality in Overweight (Panel A) and Obese (Panel B) Patients

**Panel A**

| Variable | Univariable | | | Multivariable | | |
| --- | --- | --- | --- | --- | --- | --- |
|  | **HR** | **95% CI** | **p-value** | **HR** | **95% CI** | **p-value** |
| DOAC (vs VKA) | 0.47 | 0.36, 0.62 | <0.001 | 0.52 | 0.39, 0.68 | <0.001 |
| Age ≥75 years | 3.64 | 2.59, 5.12 | <0.001 | 1.99 | 1.38, 2.88 | <0.001 |
| Women | 1.06 | 0.83, 1.35 | 0.666 |  |  |  |
| Hypertension | 1.44 | 1.01, 2.07 | 0.046 | 1.20 | 0.82, 1.77 | 0.346 |
| Diabetes | 1.09 | 0.81, 1.47 | 0.569 |  |  |  |
| CAD | 1.71 | 1.31, 2.25 | <0.001 | 1.54 | 1.14, 2.08 | 0.005 |
| Anaemia | 2.16 | 1.68, 2.76 | <0.001 | 1.31 | 1.00, 1.71 | 0.046 |
| eGFR | 0.97 | 0.96, 0.97 | <0.001 | 0.98 | 0.97, 0.99 | <0.001 |
| Heart failure | 1.83 | 1.41, 2.37 | <0.001 | 1.13 | 0.85, 1.51 | 0.392 |
| Smoking | 1.20 | 0.67, 2.13 | 0.544 |  |  |  |
| Previous stroke/TIA | 1.00 | 0.73, 1.37 | 0.994 |  |  |  |
| PAD | 1.93 | 1.30, 2.85 | 0.001 | 1.62 | 1.08, 2.42 | 0.018 |
| COPD/OSAS | 2.32 | 1.72, 3.13 | <0.001 | 1.63 | 1.19, 2.23 | 0.002 |
| Antiplatelet | 1.05 | 0.73, 1.50 | 0.798 |  |  |  |
| Class 1c AAD | 0.35 | 0.18, 0.69 | 0.002 | 0.63 | 0.32, 1.24 | 0.183 |
| Amiodarone | 0.91 | 0.63, 1.31 | 0.609 |  |  |  |
| Lipid lowering therapy | 0.66 | 0.51, 0.86 | 0.002 | 0.58 | 0.44, 0.78 | <0.001 |
| RAASi | 0.65 | 0.51, 0.82 | <0.001 | 0.63 | 0.49, 0.82 | <0.001 |
| Beta blockers | 0.97 | 0.76, 1.23 | 0.781 |  |  |  |
| Calcium channel blockers | 1.17 | 0.90, 1.53 | 0.248 |  |  |  |
| Diuretics | 1.72 | 1.35, 2.19 | <0.001 | 1.01 | 0.77, 1.33 | 0.959 |
| Digoxin | 1.22 | 0.83, 1.81 | 0.316 |  |  |  |
| PPI | 1.09 | 0.85, 1.39 | 0.497 |  |  |  |

**Panel B**

|  | Univariable | | | Multivariable | | |
| --- | --- | --- | --- | --- | --- | --- |
|  | **HR** | **95% CI** | **p-value** | **HR** | **95% CI** | **p-value** |
| DOAC (vs VKA) | 0.68 | 0.48, 0.96 | 0.028 | 0.69 | 0.48, 0.98 | 0.040 |
| Age ≥75 years | 2.63 | 1.82, 3.78 | <0.001 | 1.80 | 1.19, 2.74 | 0.006 |
| Women | 1.21 | 0.87, 1.68 | 0.264 |  |  |  |
| Hypertension | 0.61 | 0.38, 0.97 | 0.038 | 0.62 | 0.38, 1.02 | 0.059 |
| Diabetes | 1.27 | 0.90, 1.79 | 0.169 |  |  |  |
| CAD | 1.37 | 0.90, 2.07 | 0.140 |  |  |  |
| Anaemia | 2.21 | 1.56, 3.12 | <0.001 | 1.49 | 1.03, 2.15 | 0.034 |
| eGFR | 0.98 | 0.97, 0.99 | <0.001 | 0.99 | 0.98, 1.00 | 0.004 |
| Heart failure | 2.08 | 1.48, 2.92 | <0.001 | 1.65 | 1.13, 2.40 | 0.009 |
| Smoking | 0.14 | 0.02, 1.04 | 0.054 |  |  |  |
| Previous stroke/TIA | 1.67 | 1.10, 2.53 | 0.016 | 1.51 | 0.98, 2.30 | 0.060 |
| PAD | 2.66 | 1.60, 4.42 | <0.001 | 2.20 | 1.28, 3.77 | 0.004 |
| COPD/OSAS | 1.69 | 1.15, 2.50 | 0.008 | 1.29 | 0.85, 1.95 | 0.235 |
| Antiplatelet | 1.64 | 1.05, 2.57 | 0.030 | 1.12 | 0.69, 1.83 | 0.637 |
| Class 1c AAD | 0.35 | 0.13, 0.96 | 0.040 | 0.37 | 0.12, 1.17 | 0.090 |
| Amiodarone | 1.03 | 0.63, 1.70 | 0.893 |  |  |  |
| Lipid lowering therapy | 0.91 | 0.65, 1.28 | 0.588 |  |  |  |
| RAASi | 0.70 | 0.50, 0.98 | 0.038 | 0.73 | 0.51, 1.05 | 0.092 |
| Beta blockers | 0.95 | 0.68, 1.33 | 0.776 |  |  |  |
| Calcium channel blockers | 0.94 | 0.64, 1.37 | 0.743 |  |  |  |
| Diuretics | 1.52 | 1.09, 2.13 | 0.014 | 1.05 | 0.73, 1.51 | 0.802 |
| Digoxin | 0.81 | 0.44, 1.50 | 0.506 |  |  |  |
| PPI | 1.17 | 0.83, 1.63 | 0.372 |  |  |  |

*AAD: anti-arrhythmic drugs; AF: Atrial Fibrillation; BMI: body mass index; CAD: coronary artery disease; CI: Confidence Interval; COPD/OSAS: chronic obstructive pulmonary disease/obstructive sleep apnoea syndrome; DOAC: direct oral anticoagulants; eGFR: estimated glomerular filtration rate; HR: Hazard Ratio; PAD: peripheral artery disease; PPI: proton pump inhibitors; RAASi: renin-angiotensin-aldosterone inhibitors; TIA: transient ischaemic attack; VKA: vitamin K antagonist.*

## Supplementary Table 7. Univariable and multivariable Fine–Gray Competing Risk Models for Predictors of Cardiovascular Events in Overweight (Panel A) and Obese (Panel B) Patients

**Panel A**

|  | Univariable | | | Multivariable | | |
| --- | --- | --- | --- | --- | --- | --- |
|  | **sHR** | **95% CI** | **p-value** | **sHR** | **95% CI** | **p-value** |
| DOAC (vs VKA) | 0.62 | 0.49, 0.78 | <0.001 | 0.67 | 0.53, 0.86 | 0.002 |
| Age ≥75 years | 3.08 | 2.29, 4.14 | <0.001 | 1.72 | 1.25, 2.38 | <0.001 |
| Women | 1.02 | 0.81, 1.27 | 0.878 |  |  |  |
| Hypertension | 1.37 | 0.99, 1.90 | 0.055 |  |  |  |
| Diabetes | 1.20 | 0.92, 1.57 | 0.177 |  |  |  |
| CAD | 1.78 | 1.39, 2.27 | <0.001 | 1.62 | 1.23, 2.15 | <0.001 |
| Anaemia | 1.93 | 1.53, 2.43 | <0.001 | 1.24 | 0.97, 1.59 | 0.086 |
| eGFR | 0.97 | 0.96, 0.97 | <0.001 | 0.98 | 0.97, 0.98 | <0.001 |
| Heart failure | 1.78 | 1.40, 2.26 | <0.001 | 1.15 | 0.88, 1.50 | 0.295 |
| Smoking | 1.28 | 0.76, 2.16 | 0.346 |  |  |  |
| Previous stroke/TIA | 1.01 | 0.76, 1.35 | 0.946 |  |  |  |
| PAD | 2.07 | 1.45, 2.94 | <0.001 | 1.73 | 1.21, 2.48 | 0.003 |
| COPD/OSAS | 2.08 | 1.56, 2.77 | <0.001 | 1.51 | 1.12, 2.04 | 0.006 |
| Antiplatelet | 1.30 | 0.95, 1.77 | 0.096 |  |  |  |
| Class 1c AAD | 0.40 | 0.23, 0.72 | 0.002 | 0.66 | 0.37, 1.18 | 0.159 |
| Amiodarone | 0.91 | 0.65, 1.28 | 0.590 |  |  |  |
| Lipid lowering therapy | 0.76 | 0.60, 0.96 | 0.021 | 0.65 | 0.50, 0.84 | 0.001 |
| RAASi | 0.75 | 0.60, 0.94 | 0.012 | 0.75 | 0.60, 0.94 | 0.014 |
| Beta blockers | 0.88 | 0.70, 1.10 | 0.249 |  |  |  |
| Calcium channel blockers | 1.09 | 0.85, 1.40 | 0.490 |  |  |  |
| Diuretics | 1.58 | 1.26, 1.97 | <0.001 | 0.94 | 0.73, 1.21 | 0.653 |
| Digoxin | 1.09 | 0.75, 1.59 | 0.641 |  |  |  |
| PPI | 1.00 | 0.79, 1.25 | 0.977 |  |  |  |

**Panel B**

| Variable | Univariable | | | Multivariable | | |
| --- | --- | --- | --- | --- | --- | --- |
|  | **sHR** | **95% CI** | **p-value** | **sHR** | **95% CI** | **p-value** |
| DOAC | 0.80 | 0.58, 1.09 | 0.153 |  |  |  |
| Age ≥75 years | 2.24 | 1.62, 3.11 | <0.001 | 1.47 | 1.01, 2.12 | 0.042 |
| Women | 1.13 | 0.83, 1.53 | 0.436 |  |  |  |
| Hypertension | 0.66 | 0.42, 1.03 | 0.065 |  |  |  |
| Diabetes | 1.26 | 0.92, 1.72 | 0.155 |  |  |  |
| CAD | 1.39 | 0.95, 2.03 | 0.093 |  |  |  |
| Anaemia | 2.00 | 1.45, 2.77 | <0.001 | 1.49 | 1.06, 2.09 | 0.020 |
| eGFR | 0.98 | 0.98, 0.99 | <0.001 | 0.99 | 0.98, 1.00 | 0.001 |
| Heart failure | 1.85 | 1.35, 2.54 | <0.001 | 1.47 | 1.05, 2.06 | 0.025 |
| Smoking | 0.50 | 0.19, 1.35 | 0.170 |  |  |  |
| Previous stroke/TIA | 1.76 | 1.21, 2.57 | 0.003 | 1.62 | 1.10, 2.38 | 0.014 |
| PAD | 2.38 | 1.46, 3.88 | <0.001 | 1.88 | 1.12, 3.16 | 0.016 |
| COPD/OSAS | 1.71 | 1.20, 2.46 | 0.003 | 1.29 | 0.88, 1.88 | 0.191 |
| Antiplatelet | 1.73 | 1.15, 2.60 | 0.009 | 1.24 | 0.81, 1.91 | 0.326 |
| Class 1c AAD | 0.45 | 0.20, 1.02 | 0.057 | 0.48 | 0.20, 1.17 | 0.106 |
| Amiodarone | 0.92 | 0.57, 1.49 | 0.734 |  |  |  |
| Lipid lowering therapy | 0.93 | 0.68, 1.27 | 0.662 |  |  |  |
| RAASi | 0.78 | 0.57, 1.07 | 0.122 |  |  |  |
| Beta blockers | 0.87 | 0.64, 1.18 | 0.369 |  |  |  |
| Calcium channel blockers | 0.98 | 0.70, 1.38 | 0.907 |  |  |  |
| Diuretics | 1.33 | 0.98, 1.81 | 0.065 |  |  |  |
| Digoxin | 0.81 | 0.46, 1.43 | 0.468 |  |  |  |
| PPI | 1.04 | 0.76, 1.43 | 0.790 |  |  |  |

*AAD: anti-arrhythmic drugs; AF: Atrial Fibrillation; BMI: body mass index; CAD: coronary artery disease; CI: Confidence Interval; COPD/OSAS: chronic obstructive pulmonary disease/obstructive sleep apnoea syndrome; DOAC: direct oral anticoagulants; eGFR: estimated glomerular filtration rate; PAD: peripheral artery disease; PPI: proton pump inhibitors; RAASi: renin-angiotensin-aldosterone inhibitors; sHR: Subdistribution Hazard Ratio; TIA: transient ischaemic attack; VKA: vitamin K antagonist.*

## Supplementary Table 8. TTR-Stratified Analysis of Anticoagulant Users; Multivariable Cox Regression for All-Cause Mortality and Fine-Gray Model for Cardiovascular Events (Panel A: TTR ≥60%; Panel B: TTR <60%).

**Panel A**

| **Variable** | **All-cause mortality** | | | **CVEs** | | |
| --- | --- | --- | --- | --- | --- | --- |
|  | **HR** | **95% CI** | **p-value** | **sHR** | **95% CI** | **p-value** |
| **DOAC (vs VKA)** | 0.47 | 0.35, 0.63 | <0.001 | 0.64 | 0.49, 0.83 | <0.001 |
| **BMI Class: Obesity (Vs Overweight)** | 1.29 | 0.95, 1.76 | 0.104 | 1.3 | 0.98, 1.74 | 0.071 |
| **Interaction term: DOAC * Obesity*** | 1.32 | 0.83, 2.09 | 0.241 | 1.18 | 0.78, 1.80 | 0.440 |
| **Age (years)** | 1.09 | 1.07, 1.11 | <0.001 | 1.08 | 1.06, 1.10 | <0.001 |
| **Women** | 0.91 | 0.72, 1.14 | 0.401 | 0.87 | 0.71, 1.07 | 0.200 |
| **Hypertension** | 0.80 | 0.58, 1.09 | 0.161 | 0.83 | 0.63, 1.09 | 0.170 |
| **Diabetes** | 1.21 | 0.94, 1.55 | 0.139 | 1.23 | 0.97, 1.55 | 0.089 |
| **CAD** | 1.16 | 0.89, 1.52 | 0.281 | 1.23 | 0.96, 1.57 | 0.099 |
| **PAF** | 0.90 | 0.70, 1.16 | 0.410 | 1.00 | 0.80, 1.25 | 0.980 |
| **Anaemia** | 1.38 | 1.09, 1.74 | 0.008 | 1.24 | 1.00, 1.55 | 0.052 |
| **eGFR** | 0.99 | 0.99, 1.00 | 0.011 | 0.99 | 0.98, 1.00 | 0.004 |
| **Heart Failure** | 1.28 | 1.01, 1.63 | 0.045 | 1.24 | 0.99, 1.56 | 0.063 |
| **PAD** | 1.62 | 1.13, 2.32 | 0.008 | 1.64 | 1.18, 2.29 | 0.003 |
| **COPD/OSAS** | 1.66 | 1.27, 2.17 | <0.001 | 1.56 | 1.20, 2.03 | 0.001 |

**Panel B**

| **Variable** | **All-cause mortality** | | | **CVEs** | | |
| --- | --- | --- | --- | --- | --- | --- |
|  | **HR** | **95% CI** | **p-value** | **sHR** | **95% CI** | **p-value** |
| **DOAC (vs VKA)** | 0.32 | 0.23, 0.44 | <0.001 | 0.43 | 0.33, 0.58 | <0.001 |
| **BMI Class: Obesity (Vs Overweight)** | 0.95 | 0.65, 1.38 | 0.770 | 1.00 | 0.70, 1.41 | 0.980 |
| **Interaction term: DOAC * Obesity*** | 1.74 | 1.05, 2.88 | 0.031 | 1.48 | 0.94, 2.33 | 0.093 |
| **Age (years)** | 1.08 | 1.06, 1.10 | <0.001 | 1.07 | 1.05, 1.09 | <0.001 |
| **Women** | 0.92 | 0.71, 1.18 | 0.503 | 0.89 | 0.71, 1.11 | 0.310 |
| **Hypertension** | 0.90 | 0.63, 1.30 | 0.589 | 0.93 | 0.67, 1.30 | 0.680 |
| **Diabetes** | 0.85 | 0.64, 1.13 | 0.270 | 0.98 | 0.76, 1.26 | 0.850 |
| **CAD** | 1.17 | 0.87, 1.58 | 0.288 | 1.32 | 1.02, 1.71 | 0.036 |
| **PAF** | 0.83 | 0.63, 1.09 | 0.181 | 1.00 | 0.79, 1.26 | >0.999 |
| **Anaemia** | 1.53 | 1.18, 1.98 | 0.001 | 1.36 | 1.08, 1.73 | 0.010 |
| **eGFR** | 1.00 | 0.99, 1.00 | 0.288 | 1.00 | 0.99, 1.00 | 0.160 |
| **Heart Failure** | 1.22 | 0.94, 1.58 | 0.142 | 1.16 | 0.91, 1.48 | 0.230 |
| **PAD** | 1.72 | 1.18, 2.50 | 0.005 | 1.68 | 1.17, 2.40 | 0.005 |
| **COPD/OSAS** | 1.64 | 1.23, 2.19 | <0.001 | 1.55 | 1.17, 2.04 | 0.002 |

**The reported interaction term quantifies the relative modification of DOAC’s effect in obese patients compared to non-obese. The real HR for DOAC in obese patients will be obtained by multiplying the main DOAC effect by the interaction term.*

*BMI: body mass index; CAD: coronary artery disease, COPD/OSAS: chronic obstructive pulmonary disease/obstructive sleep apnoea syndrome; DOAC: direct oral anticoagulants; eGFR: estimated glomerular filtration rate; PAF: Paroxysmal Atrial Fibrillation; PAD: peripheral artery disease; PPI: proton pump inhibitors; RAASi: renin-angiotensin-aldosterone inhibitors; TIA: transient ischaemic attack; VKA: vitamin K antagonist.*

## Supplementary Table 9. Univariable Cox regression analysis of direct oral anticoagulant use (compared to warfarin) on all-cause mortality (Panel A) and Fine-Gray analysis for cardiovascular events (Panel B) according to obesity degree.

| **Panel A –** DOAC *vs VKA* – all-cause of death | **p-value** | **HR** | **95.0% CI** | |
| --- | --- | --- | --- | --- |
|  |  |  | **Low** | **High** |
| Overweight | <0.001 | 0.47 | 0.36 | 0.62 |
| 1st degree obesity | 0.030 | 0.64 | 0.43 | 0.96 |
| 2nd degree obesity | 0.573 | 0.80 | 0.36 | 1.76 |
| 3rd degree obesity | 0.809 | 0.87 | 0.27 | 2.78 |
| **Panel B -** DOAC *vs VKA* – cardiovascular events | **p-value** | **sHR** | **95.0% CI** | |
|  |  |  | **Low** | **High** |
| Overweight | <0.001 | 0.62 | 0.49 | 0.79 |
| 1st degree obesity | 0.282 | 0.82 | 0.58 | 1.18 |
| 2nd degree obesity | 0.351 | 0.69 | 0.32 | 1.49 |
| 3rd degree obesity | 0.809 | 0.87 | 0.27 | 2.78 |

*DOAC: direct oral anticoagulants, HR: Hazard Ratio, sHR: subdistribution Hazard Ratio, VKA: vitamin K antagonist*

## Supplementary Figure 1. Geographic distribution of patients of START registry with BMI >25 across Italian regions.


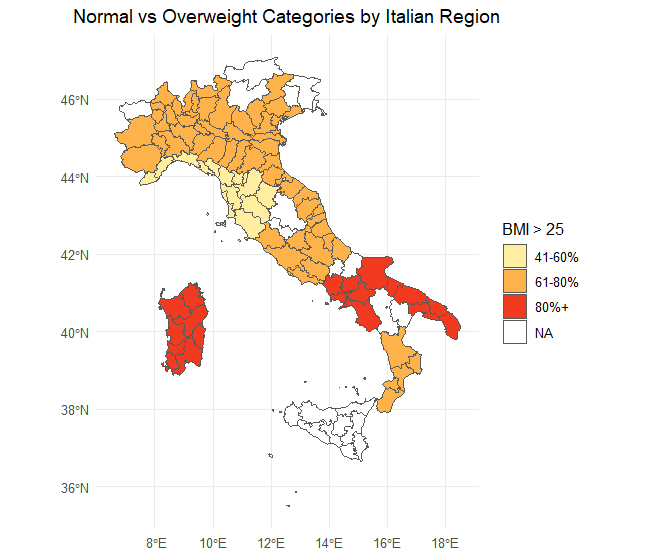


| **Italian Region** | **BMI >25 [N (%)]** | **Total Patients** |
| --- | --- | --- |
| Lombardia | 2,744 (61.3%) | 4,475 |
| Toscana | 1,015 (59.1%) | 1,716 |
| Puglia | 655 (81.1%) | 808 |
| Veneto | 526 (65.2%) | 807 |
| Piemonte | 327 (64.5%) | 507 |
| Emilia-Romagna | 332 (66.5%) | 499 |
| Lazio | 325 (67.8%) | 479 |
| Liguria | 279 (59.7%) | 467 |
| Calabria | 24 (75%) | 32 |
| Marche | 20 (69%) | 29 |
| Other | 287 (65.2%) | 440 |
| **Total** | **6,534 (63.7%)** | **10,259** |

## Supplementary Figure 2. Cumulative incidence function of CVE by anticoagulant type


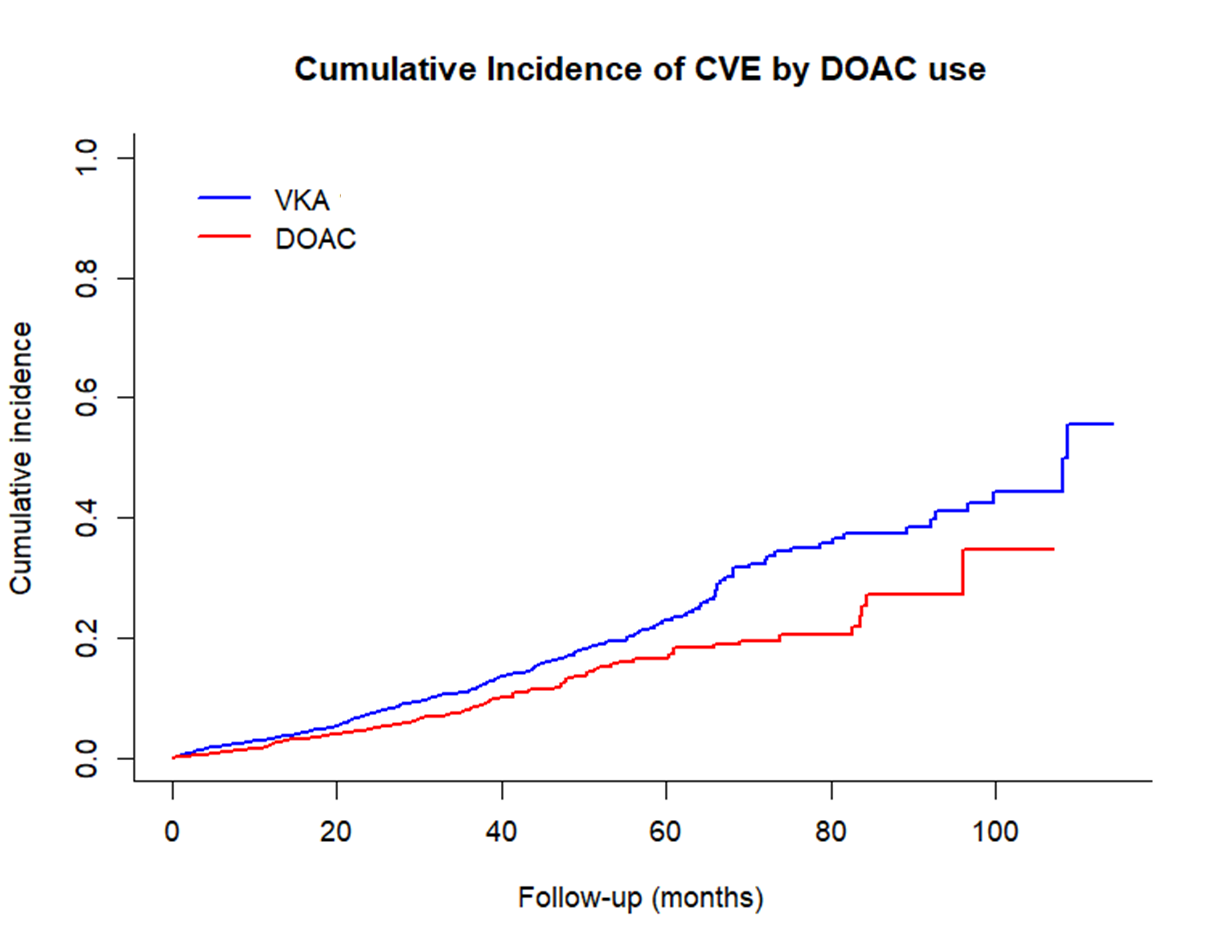


*DOAC: Direct Oral Anticoagulant; VKA: Vitamin K Antagonist.*

## Supplementary Figure 3. Association of Body Mass Index as continuous variable with all-cause mortality as modelled by restricted cubic splines regression analysis.


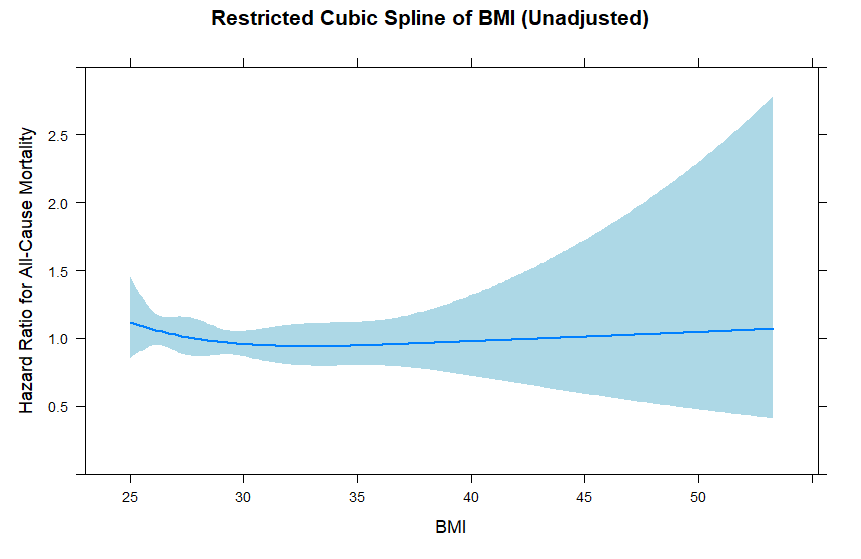


*BMI: Body Mass Index*
